# A fast all-optical 3D photoacoustic scanner for clinical vascular imaging

**DOI:** 10.1038/s41551-024-01247-x

**Published:** 2024-09-30

**Authors:** N. T. Huynh, E. Zhang, O. Francies, F. Kuklis, T. Allen, J. Zhu, O. Abeyakoon, F. Lucka, M. Betcke, J. Jaros, S. Arridge, B. Cox, A. A. Plumb, P. Beard

**Affiliations:** 1 Department of Medical Physics and Biomedical Engineering, University College London, London, UK; 2 Wellcome/EPSRC Centre for Interventional and Surgical Sciences, University College London, London, UK; 3 University College London Hospital NHS Foundation Trust, London, UK; 4 Faculty of Information Technology, Brno University of Technology, Brno, Czech Republic; 5 Centrum Wiskunde & Informatica, Amsterdam, the Netherlands; 6 Department of Computer Science, University College London, London, UK; 7 Present Address: Imperial College Healthcare NHS Trust, London, UK

**Keywords:** Translational research, Vascular diseases

## Abstract

The clinical assessment of microvascular pathologies (in diabetes and in inflammatory skin diseases, for example) requires the visualization of superficial vascular anatomy. Photoacoustic tomography (PAT) scanners based on an all-optical Fabry–Perot ultrasound sensor can provide highly detailed 3D microvascular images, but minutes-long acquisition times have precluded their clinical use. Here we show that scan times can be reduced to a few seconds and even hundreds of milliseconds by parallelizing the optical architecture of the sensor readout, by using excitation lasers with high pulse-repetition frequencies and by exploiting compressed sensing. A PAT scanner with such fast acquisition minimizes motion-related artefacts and allows for the volumetric visualization of individual arterioles, venules, venous valves and millimetre-scale arteries and veins to depths approaching 15 mm, as well as for dynamic 3D images of time-varying tissue perfusion and other haemodynamic events. In exploratory case studies, we used the scanner to visualize and quantify microvascular changes associated with peripheral vascular disease, skin inflammation and rheumatoid arthritis. Fast all-optical PAT may prove useful in cardiovascular medicine, oncology, dermatology and rheumatology.

## Abstract (web summary)

The scan times of all-optical photoacoustic tomography can be reduced to a few seconds, allowing for the visualization of microvascular changes, as shown in patients with peripheral vascular disease, skin inflammation or rheumatoid arthritis.

## Main

Visualizing the microvasculature to sub-cm depths in tissue is required for the effective clinical management of a wide range of vascular abnormalities, including those associated with skin cancers, dermatological conditions^1^, the diabetic foot^2^ and superficial soft-tissue damage such as burns or ulcers^3^. Optical imaging techniques offer promise on account of their ability to visualize vascular anatomy, oxygenation and flow^4,5^. However, strong optical scattering by tissue limits penetration depth or spatial resolution. Conventional light microscopy, optical coherence tomography and other methods that rely on unscattered ballistic photons can provide capillary-level microvascular images but only to sub-mm depths. Laser Doppler or speckle contrast imaging techniques that exploit diffuse or semi-ballistic photons provide greater penetration depth. However, optical scattering limits spatial resolution, resulting in spatially averaged images that do not reveal microvascular architecture at the level of individual microvessels. Ultrasound (US) imaging can overcome the depth/resolution limitations of optical techniques but presents other challenges. Conventional non-contrast clinical Doppler US yields poor microvascular contrast with emerging ultrafast plane-wave US^6,7^, offering higher sensitivity. However, both rely on the detection of moving blood, thus limiting their sensitivity to slowly moving or static blood and precluding the measurement of blood oxygenation.

Photoacoustic imaging provides an alternative that can address the limitations of conventional optical and ultrasound imaging^8–12^. In its most common implementation, referred to herein as widefield photoacoustic tomography (PAT)^8^, a large-area pulsed laser beam flood-illuminates the tissue. Optical absorption by haemoglobin produces impulsive heating and the subsequent generation of broadband ultrasound waves. By detecting these waves at the skin surface, an image of the vasculature can be reconstructed. Since ultrasound waves are scattered in tissue much less than photons, PAT avoids the range-resolution limitations that afflict optical methods: cm-scale penetration depths with depth-dependent spatial resolution ranging from tens to hundreds of micrometres are achievable. Moreover, unlike US, PAT directly detects the haemoglobin in blood rather than relying on blood flow as a surrogate vascular marker. It therefore offers the prospect of visualizing small vessels characterized by low blood flow otherwise indistinguishable with US, as well as the spectroscopic measurement of blood oxygenation^13^. These attributes have led to PAT being investigated for the clinical assessment of microvascular changes associated with cancer^14,15^, cardiovascular disease^16,17^, diabetes^18,19^, inflammatory conditions^20–23^ and soft-tissue damage^24,25^.

Despite its promise, the practical implementation of PAT presents non-trivial instrumentation-related challenges. These become particularly acute when imaging superficial vascular anatomy to sub-cm depths. For such shallow depths, frequency-dependent acoustic attenuation is modest, resulting in broadband photoacoustic signals with a frequency content that extends to several tens of MHz. Accurately recording the photoacoustic wavefield thus requires a commensurately wide detection bandwidth, fine spatial sampling on a tens of microns scale to satisfy spatial Nyquist and sub-100 µm element sizes. A transparent detector is also desirable to permit delivery of the excitation laser beam through the detector. Traditional piezoelectric ultrasound detectors are most commonly used in PAT scanners but tend to have an insufficiently broadband frequency response, offer poor sensitivity for sub-100 µm element sizes and are optically opaque. Moreover, for clinically acceptable three-dimensional (3D) frame rates, mechanical scanning is undesirable, necessitating a 2D detector array. For a planar aperture of 1 cm^2^ and a 10 MHz upper detection frequency, such an array would require >10^4^ elements to satisfy spatial Nyquist. Packing this number of elements within a 1 cm^2^ footprint and achieving the necessary wideband high detection sensitivity for PAT is currently unachievable with conventional piezoelectric ultrasound detection technology. The need for high element densities can be relaxed when using a spherically shaped detector array^26^ but at the cost of increased acoustic propagation distance and a physically larger array footprint. Acoustic-resolution photoacoustic microscopy (AR-PAM)^27,28^ is an alternative high-resolution photoacoustic imaging mode. However, it relies on mechanically scanning a focused detector, resulting in long acquisition times (typically >1 min) and the fixed focal depth of the detector limits the penetration depth range.

Ultrasound detectors based on optically resonant structures have the potential to overcome the limitations of piezoelectric receivers on account of their wide bandwidth, small element size, high sensitivity and optical transparency^29–37^. However, few have made the transition from the laboratory to practical in vivo PAT imaging tool. One exception is the Fabry–Perot (FP) polymer film ultrasound sensor which has been used in a range of 3D PAT scanners^37–46^. It has been shown that some of these scanners can provide in vivo 3D PAT vascular images to sub-cm depths with image quality that is superior to conventional piezoelectric-based PAT scanners. However, the clinical translation of this technology has been hindered by long acquisition times due to the sequential nature of the FP sensor readout scheme and the large number of detection points (>10^4^) required to reconstruct a high-resolution 3D image. These factors conspire to produce scan times of the order of minutes. Although acceptable for imaging relatively immobile targets such as anaesthetized mice, routine clinical use on humans requires faster acquisition on a scale of seconds or hundreds of milliseconds to minimize motion-related artefacts.

In the current work, we demonstrate a practical FP-based scanner that can meet the clinical need for fast acquisition by overcoming the slow speed of early generation systems. By exploiting a parallelized sensor readout scheme, high pulse repetition frequency (PRF) excitation lasers and compressed sensing techniques, significantly faster acquisition has been achieved. We show that scan times of a few seconds or even a few hundred ms are now possible, enabling high-fidelity 3D images without motion artefacts to be acquired repeatably in humans. In addition, dynamic 3D imaging is now possible, allowing real-time probe placement and visualization of dynamic physiological events. The increase in acquisition speed that has been achieved unlocks the clinical potential of the technology. We illustrate this by evaluating the scanner on volunteers and hospital patients and showing that it can provide detailed 3D vascular images at a variety of anatomical locations and can visualize microvascular changes associated with diabetes, skin inflammation and rheumatoid arthritis. These demonstrations illustrate the potential of the technology as a tool for the clinical assessment of diseases associated with superficial vascular pathologies.

## Results

### Fabry–Perot planar PAT scanner

**Fig. 1:**
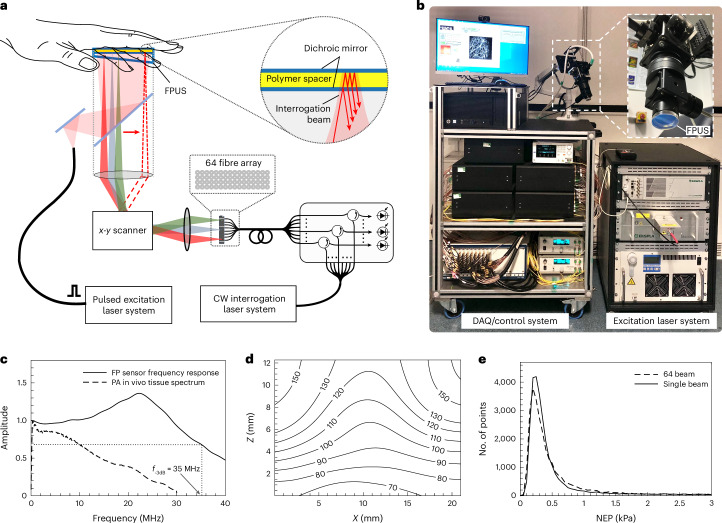
Multibeam FP-based PAT scanner. **a**, Schematic showing scanner read-out architecture and FP ultrasound sensor (FPUS) structure (see Supplementary Fig. 2 for *x*-*y* scanner schematic). **b**, Photograph of scanner showing cart-based acquisition system, imaging head (inset) and OPO excitation laser system. **c**, Frequency response of FP sensor with 24.6 µm FPI spacer (*f*_-3dB_ = 35 MHz) and acoustic frequency spectrum of photoacoustic signals generated in vivo in human palm (see Methods). **d**, Lateral spatial resolution in microns for scan step size d*x* = d*y* = 108 µm (see also Supplementary Figs. 3 and 4). **e**, NEP distribution for 64-beam and single-beam scanners obtained from NEP measurements (over a 20 MHz measurement bandwidth) made at 34,560 spatial points over a 21 × 19.5 mm^2^ scan area (see Methods): the modal NEPs are 0.2 kPa and 0.25 kPa, respectively. The FWHM of both NEP distributions are 0.25 kPa. Supplementary Fig. 9 illustrates the effect of the NEP distribution on image quality. CW, continuous wave; DAQ, data acquisition.

The custom-built scanner is shown in Fig. 1. It comprises an optical parametric oscillator (OPO) excitation laser system for generating the photoacoustic waves and an FP ultrasound scanner for mapping them over the surface of the skin. Figure 1a illustrates its working principles. Nanosecond laser pulses in the 700–900 nm spectral range where tissue attenuation is low are emitted by the OPO and coupled into a delivery optical fibre. The light emerges from the distal end of the fibre, producing a large-diameter (>20 mm) beam incident on the FP ultrasound sensor head, the acoustically sensitive element of which is a thin film polymer FP interferometer (FPI). Since the FPI is transparent in the 560–1,300 nm wavelength range (Supplementary Fig. 1), the excitation beam passes through the sensor and flood-illuminates the underlying tissue, thereby generating broadband ultrasound waves. These waves propagate to the FPI where they modulate its optical thickness and thus the interference between the light reflected from the two FPI mirrors, resulting in a modulation in the reflected optical power (Supplementary Note 1). The latter is then read out by sequentially scanning an array of up to 64 focused laser beams over the surface of the sensor step-by-step, one step per excitation laser pulse. In this way, the spatial-temporal distribution of the incident photoacoustic wavefield can be mapped in 2D, enabling a 3D image to be reconstructed.

The maximum rectangular scan area that the scanner can provide is 21 × 19.5 mm^2^. For the step sizes of d*x* = d*y* = 108 µm and d*x* = d*y* = 54 µm used in this study, this enables the synthesis of arrays of 34,560 and 138,240 detection points, respectively. Dense spatial sampling on this scale is a prerequisite for short-range high-resolution PAT and achievable with the FP sensor by virtue of the optical nature of its readout scheme but unattainable with current conventional ultrasound detection technology. As shown in Fig. 1c, the sensor also provides the necessarily wide frequency response (50 kHz–35 MHz) to capture the broadband frequency content of photoacoustic signals generated in tissue, a requirement that is also challenging to meet using conventional methods. The combination of dense spatial sampling and wide bandwidth that the system provides yields near-acoustic diffraction-limited spatial resolution; at the centre of the scan area, the lateral resolution varies from 60 µm to ~120 µm over the depth range 1 mm to 12 mm (Fig. 1d).

The measured vertical resolution was found to be spatially invariant at 45 µm over the field of view (FOV). The FP sensor also offers high sensitivity with small element size. Figure 1e shows a histogram of the noise equivalent pressure (NEP) distribution over a scan area of 21 × 19.5 mm^2^. The modal NEP is 0.2 kPa (over a 20 MHz measurement bandwidth) and achieved using an interrogation beam spot diameter (1/e^2^) of 49 µm which, to a first approximation, corresponds to the acoustic element size^47^. By comparison, the sensitivity of an equivalent-sized broadband PVDF piezoelectric receiver would be more than two orders of magnitude lower, a consequence of the reduction in piezoelectric sensitivity that arises with decreasing element area^32^.

### Accelerated acquisition implementation

The key technical advance that has been made relates to reducing acquisition time. To achieve this, two strategies were variously employed. The first involved increasing the A-line acquisition rate by parallelizing the sensor readout and using high-PRF excitation lasers. In the second, a compressed sensing approach was adopted, permitting subsampling of the acoustic detection aperture and thus a reduction in the total number of A-lines required to form an image.

The parallelization of the FP sensor readout was achieved using a multibeam scanner that scans an array of *N* interrogation beams across the sensor; *N* = 16 or *N* = 64, with the latter most commonly used and assumed in the following description. As shown in Fig. 1a, the output of the fibre-coupled interrogation laser system (see Methods) is divided into 64 single-mode 9 µm core optical fibres using a passive fibre splitter. Each fibre is connected to a fibre circulator and recombined to form a fibre bundle comprising four rows of 16 fibres with a core-to-core separation of 80 µm. The divergent output of each fibre in the bundle is incident on a lens that collimates all 64 beams and brings them together to form a pivot point at the input of a 2-axis conjugate galvanometer-based scanner (Supplementary Fig. 2). The output of the scanner is focused onto the plane of the FP sensor, resulting in an array of 64 focused spots, each separated by 324 µm, incident on the sensor. The 64 beams are reflected from the sensor, back through the scanner, recoupled into their respective single-mode fibres and delivered via circulators to individual InGaAs photodiode-transimpedance amplifier units, each connected to an input of a 64-channel radio frequency (RF) digitizer. By scanning the array of 64 beams across the sensor surface and recording the acoustically induced time-varying reflected optical power modulation simultaneously on all 64 channels at each scan point, a map of the incident photoacoustic wavefield can be recorded. In all cases in this study, single-shot acquisition without signal averaging was used.

Parallelizing the detection in this way sets a specific technical challenge. To detect a signal with high sensitivity, it is necessary to tune the interrogation laser to a wavelength that aligns with the peak derivative of the FP interferometer transfer function (ITF). This wavelength is denoted the optimum bias wavelength *λ*_b_^37^. Since the interrogation laser wavelength is identical for all 64 beams, the FPI polymer spacer thickness must be sufficiently uniform such that *λ*_b_ is spatially invariant over the 6.72 mm^2^ rectangular area d*A*_b_ on the sensor occupied by the 64 focused beams. For the sensors used in this study, the ITF full-width at half-maximum (FWHM) was >0.35 nm, requiring variations in the bias wavelength d*λ*_b_ over d*A*_b_ to be less than 0.08 nm for the sensitivity to remain within ~90% of its maximum value. The corresponding thickness uniformity *dl* required to meet this condition is given by *dl* = *l*d*λ*_b_/*λ*_b_ where *l* is the spacer thickness^48^. For a spacer thickness *l* = 25 µm and *λ*_b_ = 1,550 nm, it is therefore required that *dl* < 1.3 nm over d*A*_b_, a non-trivial requirement for polymeric films. If this condition is not met, the acoustic sensitivity will vary from one beam to the next, compromising the reconstructed image signal-to-noise ratio (SNR). Spacer thickness uniformities as low as 2.4 nm over 100 mm^2^ were achieved corresponding to ~*λ*/650; for comparison, the thickness uniformity of a high-quality solid fused silica etalon rarely exceeds *λ*/100. This level of thickness uniformity comfortably surpasses the requirement for *λ*_b_ parity over d*A*_b_ as evidenced by Fig. 1e which shows that the spatial distribution of the NEP of the 64-beam scanner is comparable to that of a single-beam scanner. The latter serves as a benchmark for comparison because the bias wavelength can be optimally set at each spatial point when using a single interrogation beam, thus enabling the optimum NEP distribution to be achieved for a given FP sensor.

Fast acquisition was further enabled by using excitation lasers operating at high PRFs and optimizing the scanner control and data acquisition hardware to accommodate these PRFs. Frequency doubled Nd:YAG pumped Type 2 OPO laser systems, emitting 5 ns pulses at PRFs up to 200 Hz were used for most imaging studies (see Methods). In addition, a custom Yb fibre laser^49^ operating at a PRF of 1 kHz emitting 20 ns pulses was used to approach the maximum achievable A-line rate limited by the system hardware. The combination of parallelizing the sensor readout and operating at these relatively high PRFs enabled significant A-line rate acceleration. Depending on *N* and the PRF, A-line rates between 1,400 and 64,000 A-lines per second were achieved, compared to the 50 A-lines per second of early-generation single-beam preclinical FP scanners^43^. Reducing the scan time by subsampling the scan area to reduce the total number of scan points was also evaluated. By exploiting the data redundancy in the photoacoustic wavefield within a compressed sensing framework^50^ (see Methods), subsampling factors of 50, 25 and 12.5% were used with corresponding reductions in scan time.

The above methods have reduced the time required to acquire 3D image data sets; depending on the imaging parameters used, scan times on a scale of seconds to hundreds of ms were achieved.

### 3D in vivo vascular imaging

To demonstrate the rapid, volumetric imaging capabilities of the scanner, images of vascular anatomy were acquired using healthy adult volunteers as research participants (see Methods). To acquire an image, the probe head was positioned at the anatomical region of interest (ROI), a bolus of ultrasound gel inserted between the FP sensor and the skin to provide acoustic coupling, and the scan sequence commenced.

**Fig. 2:**
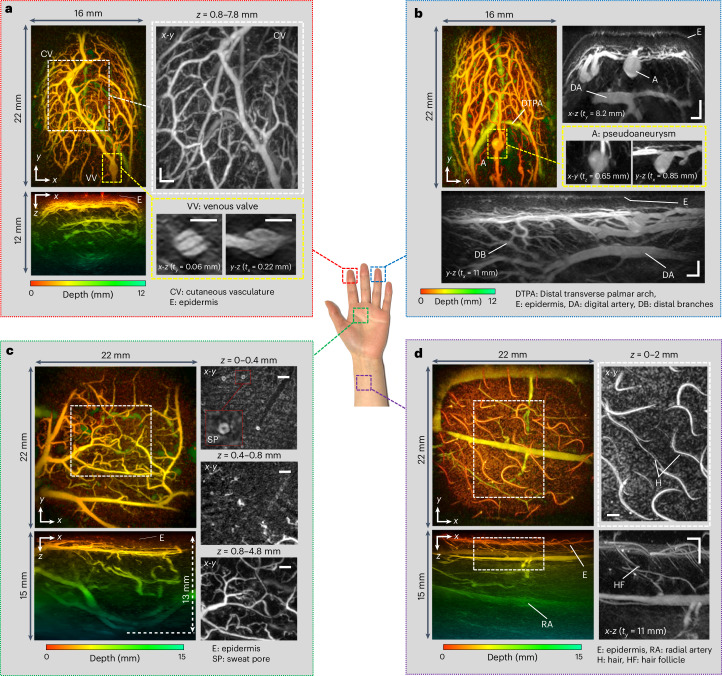
PAT images of the hand and wrist regions. **a**, Index fingertip. Left: *x*-*y* and *x*-*z* depth-to-colour encoded MIPs. Right: expanded view greyscale MIPs showing en-face view of cutaneous vasculature (CV) and cross-sectional views of a venous valve (VV). **b**, Ring fingertip. Left: *x*-*y* depth-to-colour encoded MIP. Right: expanded view greyscale MIPs showing cross-sectional view of dermal microvascular anatomy, epidermis (E), digital artery (DA) and pseudoaneurysm (A). Bottom: expanded view *y*-*z* greyscale cross-sectional MIP showing distal branches (DB) and DA. **c**, Palm region. Left: *x*-*y* and *x*-*z* depth-to-colour encoded MIPs showing vascular anatomy to a depth of 13 mm. Right: expanded view *x*-*y* en-face greyscale MIPs for three different depth ranges for region indicated by dashed white box. **d**, Wrist region. Left: *x*-*y* and *x*-*z* colour-depth encoded MIPs, the latter showing the radial artery (RA). Right: expanded view greyscale MIPs of region indicated by dashed white box showing hair (H) and hair follicles (HF). Scale bars, 1 mm. *t*_*x*_ and *t*_*y*_: slice thicknesses of *y*-*z* and *x*-*z* greyscale MIPs, respectively. Imaging parameters: *λ* = 850 nm, d*x* = d*y* = 108 µm, d*t* = 16.67 ns, PRF = 100 Hz, *N* = 64, A-line rate: 6,400 A-lines per second, scan time: *T* = 5.4 s.

Figure 2 shows maximum intensity projections (MIP) of 3D image data sets acquired at different positions on the hand and wrist. These images were reconstructed in <1 s using a *k*-space method^51^ from 34,560 photoacoustic A-lines recorded over a scan area of 21 × 19.5 mm^2^ in 108 µm steps. The PRF-limited A-line rate was 6,400 A-lines per second, resulting in a total scan time *T* = 5.4 s. By contrast, early-generation FP scanners^42,43^ would have taken several minutes to perform an identical scan.

**Extended Data Fig. 1:**
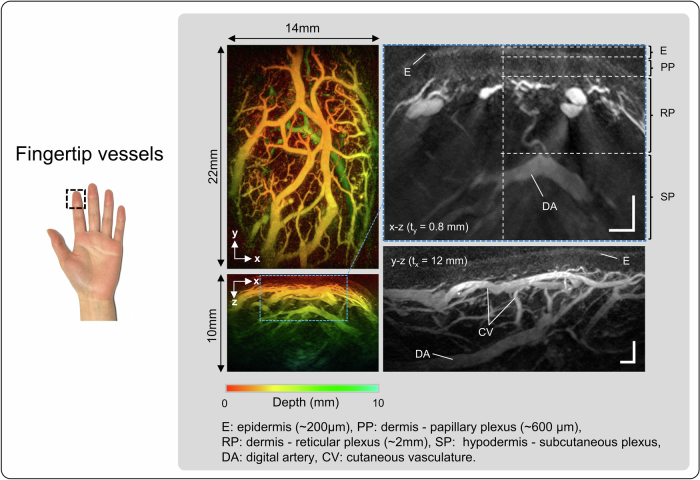
PAT image of index finger-tip vasculature. Left; x-y and x-z depth-to-colour encoded MIPs. Right, expanded view greyscale MIPs showing x-z and y-z cross sectional views. Scale bars: 1mm. tx and ty: slice thicknesses of x-z and y-z greyscale MIPs respectively. Imaging parameters: λ=850nm, dx=dy=108µm, dt=16.67ns, PRF=100Hz, N=64, A-line rate: 6400 A lines/s, scan-time: T=5.4s.

As Fig. 2 shows, the scanner provides detailed volumetric images of the microvasculature to depths approaching 15 mm. The images of the index and ring fingertips (Fig. 2a,b) show a superficial layer (E) less than 200 µm thick. This is the predominantly avascular epidermis with the contrast provided by melanin rather than haemoglobin absorption (see Extended Data Fig. 1 for additional depth-resolved visualizations). Beneath the epidermis, at a depth of ~0.5 mm, dense networks of small vessels within the superficial papillary plexus are visible. In this region, individual vessels with reconstructed lateral and vertical dimensions down to 80 µm and 50 µm, respectively, are visible. At greater depths, the larger vessels in the reticular plexus, the distal transverse palmar arch (DTPA) and the digital artery (DA) in the hypodermis can be seen (Fig. 2b).

Other visible structures include venous microvalves (VV) in the superficial papillary plexus, most evidently in the lower right *x*-*z* and *y*-*z* greyscale images in Fig. 2a. These images reveal the V-shaped structure characteristic of the internal folds that serve as the leaflets of a bicuspid venous valve, also observed in transmission electron microscopy images of resin casts of valves^52^ but not previously visualized in vivo in 3D in sub-mm venules. Another distinctive vascular structure is the berry-shaped feature protruding from a superficial vessel shown in Fig. 2b, possibly a pseudoaneurysm (A) or saccular dilation. The image of the palm and wrist regions (Fig. 2c,d) show a thicker epidermis, a less dense superficial vasculature, large vessels such as the radial artery (Fig. 2d) and greater penetration depth with discernible vascular anatomy at a depth of 13 mm (Fig. 2c). Also visible are non-vascular structures including sweat pores, skin sulci, hair and hair follicles.

**Fig. 3:**
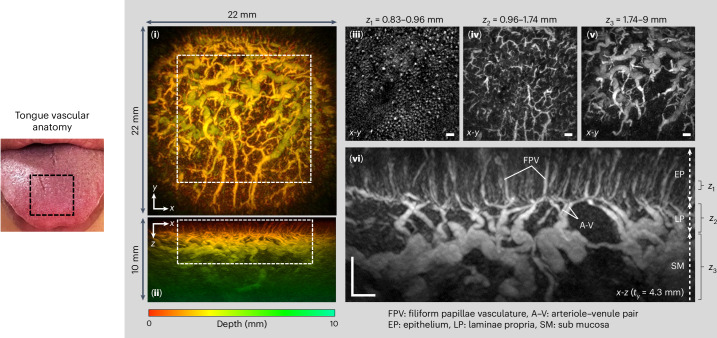
PAT image of the tongue dorsum. (**i**) *x*-*y* and (**ii**) *x*-*z* depth-to-colour encoded MIPs. Greyscale *x*-*y* MIPs of region indicated by dashed white rectangle in (**i**) for (**iii**) 0.13 mm thick slice through the epithelium (EP), *z* = 0.83–0.96 mm; (**iv**) 0.78 mm thick slice corresponding to laminae propria (LP), *z* = 0.96–1.74 mm; (**v**) 7.26 mm thick slice through submucosa (SM) and muscle, *z* = 1.742–9 mm. (**vi**) Expanded view greyscale *x*-*z* MIP of region indicated by dashed white rectangle in (**ii**) showing filiform papillae (FP) capillary loops, arteriole–venule pair (A–V) and supplying vasculature. Scale bars, 1 mm. *t*_*y*_: slice thicknesses of *x*-*z* greyscale MIP. Imaging parameters: *λ* = 875 nm, d*x* = d*y* = 108 µm, d*t* = 16.67 ns, PRF = 100 Hz, *N* = 64, A-line rate: 6,400 A-lines per second, scan time: *T* = 5.4 s.

To demonstrate the versatility of the scanner, other regions of the body that exhibit different distinctive vascular anatomy were scanned. Figure 3 shows an image of the dorsal region of the tongue illustrating the rich diversity of vascular architectures, orientations and length scales that can be visualized. The superficially located near-vertical capillaries in the filiform papillae and their supplying arteriole–venule pairs, the dense network of microvessels in the lamina propria and the larger mm-scale horizontally aligned supplying vessels in the underlying submucosa and muscle are all visible.

**Fig. 4:**
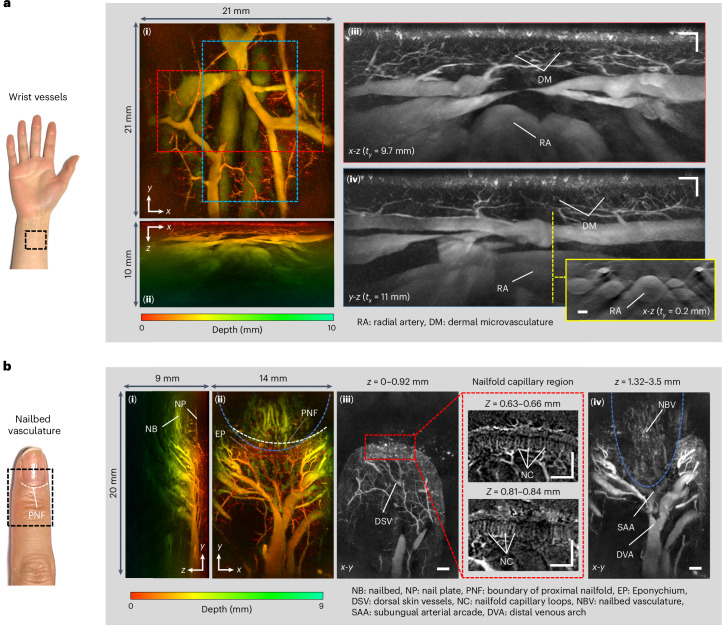
PAT images of the wrist and nailbed vasculature acquired in high-resolution scan mode. **a**, Wrist region, (**i**) *x*-*y* and (**ii**) *x*-*z* depth-to-colour encoded MIPs, (**iii**) *x*-*z* and (**iv**) *y*-*z* greyscale MIP slices of regions indicated by dashed red and blue rectangles in (**i**) showing fine dermal microvasculature (DM), radial artery (RA) and large wrist veins. Inset*:* *x*-*z* greyscale MIP showing cross-sectional view of the radial artery and adjacent veins in the plane indicated by the dashed yellow line in (**iv**). **b**, Nailfold and nailbed region of the finger, (**i**) *y*-*z* depth-to-colour encoded MIP showing dermal vasculature, nail plate (NP) and nailbed (NB); (**ii**) *x*-*y* depth-to-colour encoded MIP showing en-face view of supplying vascular tree and eponychium (EP). Dashed white line represents the boundary of the proximal nailfold (PNF). (**iii**) *x*-*y* MIP greyscale slice (*z* = 0–0.92 mm*)* revealing dorsal skin vessels (DSV) and nailfold capillary (NC) region. Right: expanded view of dashed red rectangle showing layers of nailfold capillary loops at two different depths. (**iv**) *x*-*y* MIP greyscale slice (*z* = 1.32–3.5 mm) showing nailbed vasculature, subungual arterial arcade (SAA) and distal venous arch (DVA). In (**ii**) and (**iv**), the region visualized within the dashed blue line lies beneath the nail plate. To account for the higher speed of sound in the nail, this region was reconstructed using a different sound speed (*c* = 1,650 m s^−1^) from the surrounding region (*c* = 1,547 m s^−1^), enabling improved visualization of the vasculature beneath the nail plate. Scale bars, 1 mm. *t*_*x*_ and *t*_*y*_: slice thicknesses of *y*-*z* and *x*-*z* greyscale MIPs, respectively. Imaging parameters: *λ* = 850 nm, d*x* = d*y* = 54 µm, d*t* = 16.67 ns, PRF = 100 Hz, *N* = 64, A-line rate = 6,400 A-lines per second, scan time *T* = 29 s.

**Extended Data Fig. 2:**
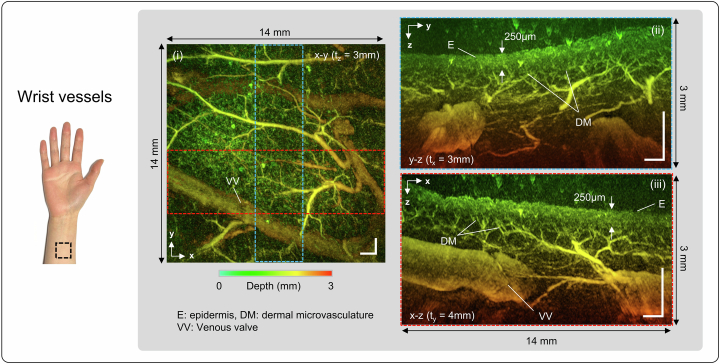
PAT image of the wrist vasculature acquired in high-resolution scan mode. (i) x-y depth-to-colour encoded MIP. (ii-iii) depth-to-colour encoded y-z and x-z MIPs of the regions denoted by the blue and red dotted line in (i). The y-z and x-z MIPs show the epidermis (E) with a thickness of 250µm, the fine dermal microvasculature (DM), a venous valve (VV) and the large wrist veins. Scale bars: 1mm. tx and ty: slice thicknesses of y-z and x-z MIPs respectively. Imaging parameters: λ=850nm, dx=dy=54µm, dt=16.67ns, PRF=100Hz, N=64, A-line rate=6400 A lines/s, scan-time T=29s.

To acquire even more detailed images, a high-resolution scan mode in which the step size was reduced to 54 µm was used. Figure 4 shows an image of the vessels in the wrist acquired in this mode. At depths below 2 mm, the fine dermal microvasculature can be seen. In this region, individual vessels with reconstructed lateral and vertical dimensions down to 45 µm and 35 µm, respectively, are visible. In addition, much larger mm-scale vessels such as the radial artery can be seen. It is notable that the interiors, as well as the edges of these vessels are clearly visualized as shown in the inset *x*-*z* MIP in Fig. 4a(iv). This is a consequence of the broad bandwidth of the FP sensor that extends down to 50 kHz, enabling the detection of the low acoustic frequencies required to accurately reconstruct mm-scale features. By contrast, images acquired by piezoelectric-based PAT scanners tend to visualize only the boundaries of large vessels^53^, a consequence of the poor sensitivity at low frequencies of the relatively narrowband piezoelectric receivers used in these systems. Extended Data Fig. 2 shows an additional high-resolution scan of the wrist region, with the imaging parameters chosen to optimally visualize the epidermis and superficial dermal vasculature. A high-resolution scan was also performed on the upper side of the finger in the nailbed area further illustrating the diversity of vascular anatomy that can be visualized. The major digital vessels such as the distal venous arch and those constituting the subungual arterial arcade can be seen. In addition, small microvascular structures, including the nailfold capillary loops which can serve as markers of conditions such as rheumatoid arthritis^54^ or Reynaulds phenomenon^55^, are visible.

A further capability facilitated by the fast acquisition now achievable is the ability of the scanner to rapidly acquire 3D images at different wavelengths. To demonstrate this, images of the wrist region at four different wavelengths were acquired (total scan time *T* = 21.6), illustrating the different spectral characteristics of melanin and arterial and venous blood (Supplementary Fig. 5).

These results show that the scanner can provide detailed 3D vascular images of comparable, if not better, quality than previous FP scanners but with acquisition times on the order of seconds rather than minutes.

### Accelerated acquisition modes

**Fig. 5:**
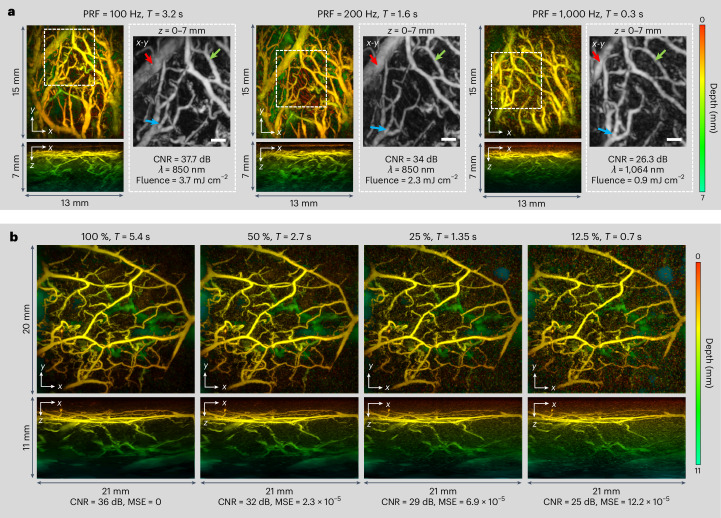
Accelerated PAT acquisition modes. **a**, Images of the vasculature acquired at PRFs of 100, 200 and 1,000 Hz. The corresponding A-line rates were 6,400, 12,800 and 64,000 A-lines per second resulting in scan times of *T* = 3.2, *T* = 1.6 and *T* = 0.3 s, respectively. The dashed white rectangles represent the region of the vasculature common to all three images; it is within this region that the CNR is estimated. The greyscale *x*-*y* MIPs show expanded views of this region, with the red, green and blue arrows depicting common blood vessels. **b**, Images acquired at *λ* = 850 nm using fully sampled data (100%) and spatially subsampled data with subsampling factors of 50, 25 and 12.5% and corresponding scan times of *T* = 5.4, *T* = 2.7, *T* = 1.35 and *T* = 0.7 s, respectively. A-line rate = 6,400 A-lines per second. Scale bars, 1 mm. Imaging parameters: d*x* = d*y*, d*x* = d*y* = 108 µm, d*t* = 16.67 ns, *N* = 64.

The detailed nature of the images shown in Figs. 2–4 suggests that acquisition speed is sufficiently high to avoid appreciable motion-related image artefacts (see Supplementary Fig. 6 for an illustration of the influence of scan time on in vivo image quality). However, even faster acquisition is desirable in some circumstances, for example, for visualizing haemodynamic events. To achieve this, images were acquired using excitation lasers operating at higher PRFs than typically used in PAT and exploiting compressed sensing principles. Figure 5a shows images of a 14 × 14 mm^2^ region on approximately the same region of the palm at three different PRFs. The images acquired at 100 Hz and 200 Hz PRFs were obtained at 875 nm using an OPO laser system, resulting in PRF-limited A-line rates of 6,400 A-lines per second and 12,800 A-lines per second and corresponding acquisition times of 3.2 s and 1.6 s, respectively. There is a reduction of 3.7 dB in image contrast to noise ratio (CNR) at 200 Hz due to the lower pulse energy available from the laser system at this PRF. To demonstrate that the scanner hardware can operate at even higher PRFs, an image was obtained using a PRF of 1 kHz provided by a custom-designed, large-core Yb fibre laser^49^ (Methods) emitting at 1,064 nm. The A-line rate achieved at this PRF was 64,000 A-lines per second, resulting in a scan time of *T* = 0.3 s. Image quality appears not to be excessively compromised, given the non-optimal excitation wavelength and low pulse energy of the laser that resulted in a fluence more than one order of magnitude lower than the maximum permissible exposure (MPE) (see Supplementary Note 2 for MPE estimates).

There is inevitably a limit to the scan speed that can be achieved by increasing the PRF. This is dictated by the response time of the galvanometer scanners, data acquisition sampling and transfer rates, the availability of high-PRF mJ-scale ns pulsed lasers and, perhaps most importantly, the average power constraints defined by the maximum permissible laser exposure for the skin. The use of spatially subsampled data within a compressed sensing framework provides an opportunity to circumvent these limitations. It is based on the notion that the spatial complexity of photoacoustic images is modest and thus, conventionally uniformly sampled photoacoustic data contain an element of redundancy^56–58^. This redundancy can be exploited to reconstruct an image without excessively compromising image CNR by randomly subsampling the scan area and employing an iterative model-based variational image reconstruction approach that imposes a sparsity constraint^50,59^. Since this image is formed from fewer measurements, the scan time *T* is reduced accordingly.

The results of using this approach (see Methods and Supplementary Note 3) are illustrated in Fig. 5b which shows images reconstructed using different subsampling factors. These images were obtained by randomly scanning the interrogation beam array in a non-overlapping fashion to acquire a fully sampled (100%) photoacoustic time-series data set with a scan time *T* = 5.4 s and image CNR = 36 dB. Thereafter, subsampled data sets were obtained by extracting the first 12.5%, 25% and 50% of the recorded data from the 100% data set with corresponding scan times of *T* = 0.7, *T* = 1.35 and *T* = 2.7 s and image CNRs of 25, 29 and 32 dB, respectively. As Fig. 5b and Supplementary Fig. 8 show, CNR scales inversely with subsampling factor, although, even when using just 12.5% of the fully sampled data, the main features of the vasculature remain visible. As Supplementary Figs. 7 and 8 show, the main compromise when using subsampled data is the reduction in image CNR that arises as the subsampling factor is increased. The degradation in spatial resolution on the other hand is somewhat modest, a consequence of the edge preserving characteristics of the TV regularization used in the image reconstruction scheme. However, there is a cost in terms of increased run time due to the need to run the forward acoustic propagation model multiple times within the iterative reconstruction scheme used; for example, the reconstruction time was up to 25 min for the images in Fig. 5b compared with <1 s for the images in Fig. 2 (see Methods). However, recent developments in the use of deep learning for iterative PAT image reconstruction^60,61^ may provide opportunities to reduce computation time.

### 2D dynamic imaging

**Fig. 6:**
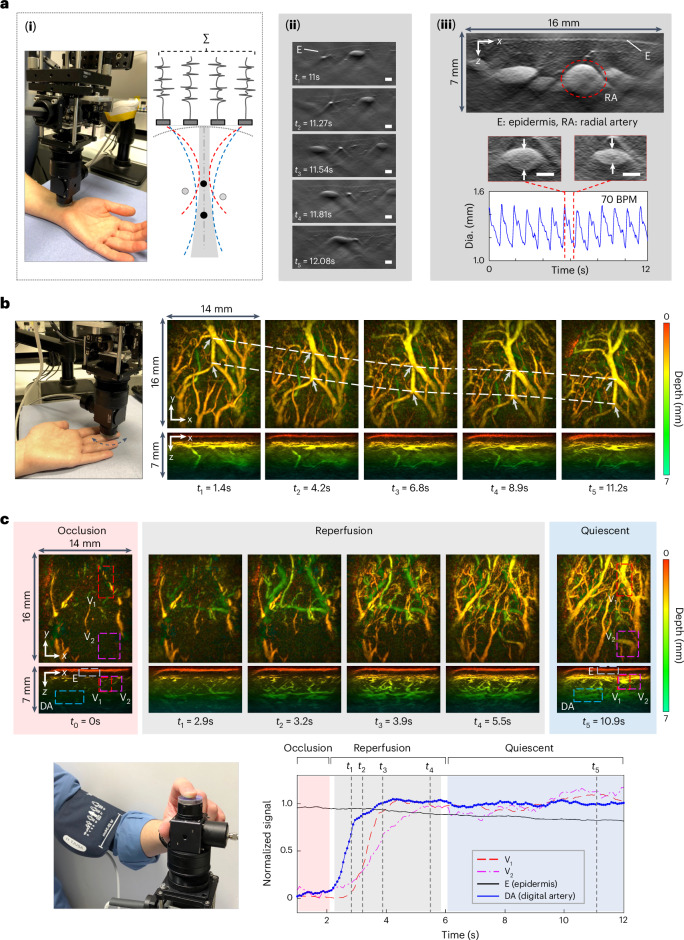
Dynamic PAT imaging. **a**, Video-rate 2D imaging. (**i**) Left: probe head positioned on wrist. Right: illustration of elevational receive beamforming (the red and blue dotted lines indicate two receive focii at different depths). (**ii**) Sequence of images of blood vessels in the wrist region acquired at different time points at a frame rate of 33 fps as probe head is moved across the skin surface from right to left; see Supplementary Video 1 for complete image sequence. (**iii**) Top: image of radial artery (RA) and adjacent veins in the wrist. Middle: two images of radial artery acquired 0.8 s apart showing change in its dimensions due to the pulsatile motion of blood flow; see Supplementary Video 2 for complete image sequence. Bottom: oscillatory time variation of radial artery size at 70 beats per minutes (BPM). Single-frame scan time *T* = 30 ms. **b**, Real-time 3D imaging. Images acquired at 6 different time points as the fingertip is translated across the probe head. The pair of dotted white lines shows the translational trajectory of the two vessel bifurcations identified by white arrows. See Supplementary Video 4 for complete image sequence. **c**, Haemodynamic response due to arterial cuff occlusion. Top: images of fingertip vasculature acquired at 5 different time points showing occlusion, reperfusion and quiescent phases. See Supplementary Video 6 for complete image sequence. Bottom: time course of vascular signal in ROIs delineated by rectangles corresponding to two vessel regions (V1 and V2), the epidermal layer (E) and the digital artery (DA). Scale bars, 1 mm. Imaging parameters: *λ* = 850 nm, PRF = 200 Hz, *N* = 64, A-line rate 12,800 lines per second, d*x* = d*y* = 162 µm, d*t* = 16.67 ns.

The system can provide a 2D video-rate imaging mode for visualizing dynamic physiological events or to rapidly search for a region of anatomical interest in real time before switching to the 3D imaging mode. To acquire a 2D image, the 4 × 16 interrogation beam array is scanned along a line on the FP sensor, with the long axis of the array aligned along the scan line. The photoacoustic signals acquired can thus be regarded as equivalent to those acquired from a 1.5D ultrasound array composed of 4 × 16 × *m* elements, where *m* is the number of scan steps along the line scan. By performing depth-dependent synthetic receive focusing in the elevation plane of this notional 1.5D array, out-of-plane photoacoustic signals are rejected and the image slice thickness minimized (Fig. 6a(i)). To demonstrate the video-rate 2D imaging capability of the system, the probe was placed on the wrist of a volunteer and translated over the skin surface while acquiring images as shown in Supplementary Video 1. Figure 6a(ii) shows a sequence of 5 frames extracted from this video corresponding to different probe positions. Several blood vessels and their change in relative lateral position as the probe is moved from right to left can be seen. To acquire these images, the interrogation beam array was scanned along a 15.55 mm long line in steps of 162 µm and an excitation laser PRF of 200 Hz was used. Hence, the A-line rate was 12,800 lines per second, resulting in a single-frame scan time of 30 ms and thus a frame rate of 33 fps.

In a second experiment, the radial artery (Fig. 6a(iii)) was imaged at 33 fps, enabling the time-varying change in its dimensions due to the pulsatile motion of the blood flow to be visualized. This is illustrated in Supplementary Video 2 with Fig. 6a(iii) showing a plot of the oscillatory variation in the minor axis of the artery at a frequency corresponding to the physiologically normal 70 beats per minute. In the examples shown in Fig. 6a, the photoacoustic time-series data were downloaded following the scan and the images reconstructed and visualized offline. Supplementary Video 3 shows an example where images of the skin vasculature were reconstructed and visualized online in real time as the probe head was translated across the skin surface. This incurs a modest time penalty compared with offline reconstruction due to the limited data transfer speed from the memory of the RF digitizer card, resulting in a reduced frame rate of 25 fps.

### 3D dynamic imaging

To illustrate the dynamic 3D imaging capability of the system, two experiments were undertaken. In the first, a sequence of images was acquired as the probe head was translated over the surface of the fingertip as shown in Supplementary Video 4. Five representative frames reconstructed from the data used to produce this Video are shown in Fig. 6b. They correspond to different time points during the motion of the probe, with the dashed white lines showing the translational trajectory of a pair of vessel bifurcations (white arrows). To acquire the data for these images, the scan area was continuously scanned over a period of 12 s according to a predetermined randomized spatial pattern, acquiring a total of 153,600 A-lines. Each of the images in Fig. 6b was reconstructed from 7,680 A-lines acquired in 0.6 s. To provide a smooth dynamic visualization in Supplementary Video 4, a sliding window advanced in increments of 10% was applied to the entire measured photoacoustic data set. Thus, the first frame was reconstructed from the first 7,680 A-lines. To reconstruct the second frame, the first 768 A-lines (the first 10%) were replaced by the A-lines numbered 7,681 to 8,449 (the next 10%) and so on, with the window advancing incrementally by 10% until it reaches the end of the complete 153,600 A-line measured data set. Supplementary Video 4 therefore comprises 200 frames acquired over 12 s with a frame rate of 16.7 fps; the latter is termed the ‘refresh frame rate’ since each frame is an update of the previous one with just 10% new A-line data rather than being formed from an entirely new set of A-lines. In this example, the reconstruction and visualization of the images were performed offline following acquisition of the complete data set. However, real-time dynamic imaging can also be performed online in 3D to facilitate ROI localization and probe positioning. This is presented in Supplementary Video 5 which shows the probe head being translated across the palm but in this case, the 3D images are reconstructed and displayed in real time using a 50% sliding window with a refresh frame rate of 3 fps.

In the second experiment, an invoked haemodynamic response was visualized. This was achieved by placing an inflatable cuff around the upper arm of a volunteer and inflating it to a pressure of 230 mmHg for 5 s to produce an arterial occlusion, thereby temporarily reducing perfusion in the downstream digital microvasculature. Following the release of the cuff, the vasculature is rapidly reperfused, achieving a steady state thereafter. These changes were visualized by placing the forefinger tip on the sensor and continuously randomly scanning the sensor for a period of 12 s. Supplementary Video 6 shows the complete image data set, reconstructed and visualized with a 16.7 fps refresh frame rate using the previously described 10% sliding window method. Figure 6c shows a sequence of six image frames corresponding to the occlusion, reperfusion and quiescent periods. In addition, the temporal evolution of the mean image intensity within ROIs corresponding to the epidermis (E), two regions of the vasculature (V_1_ and V_2_) and the digital artery (DA) are plotted. These results show that during the occlusion period, the contrast exhibited by the vasculature is weak. When the cuff is released, rapid reperfusion occurs and the contrast increases, attaining its normal level within 2–3 s. The rate of increase is similar for all three vascular ROIs (V_1_, V_2_ and DA), although the onset time for each is different; the signal corresponding to digital artery (DA) is the first to increase as expected since it is the arterial flow that resumes first, followed by V_1_ and V_2_. Notably, the signal corresponding to the epidermis is relatively constant over time, which is consistent with the assumption that the contrast in this region is predominantly non-vascular, originating from dermal melanin rather than blood. The ability to visualize haemodynamic events in this way could enable the study of time-varying physiological responses such as post-occlusive reperfusion, pressure-induced vasodilation or thermal reflex events^62^. This could potentially be utilized for the clinical assessment of diabetes and other diseases associated with impaired microvascular reactivity^2,63^.

### Exploratory clinical case studies

To illustrate how the scanner could be used clinically, exploratory case studies on patients with suspected vascular changes associated with peripheral vascular disease, skin inflammation and rheumatoid arthritis were undertaken (see Methods). These are preliminary studies not intended to provide a clinical validation of the technology but to illustrate potential areas of application that warrant future, more comprehensive clinical studies. Moreover, they demonstrate the feasibility of using the scanner on a real-world patient cohort where imaging is more challenging due to frailty, co-morbidity or pain that may limit their ability to tolerate prolonged scanning times.

#### Peripheral vascular disease

**Fig. 7:**
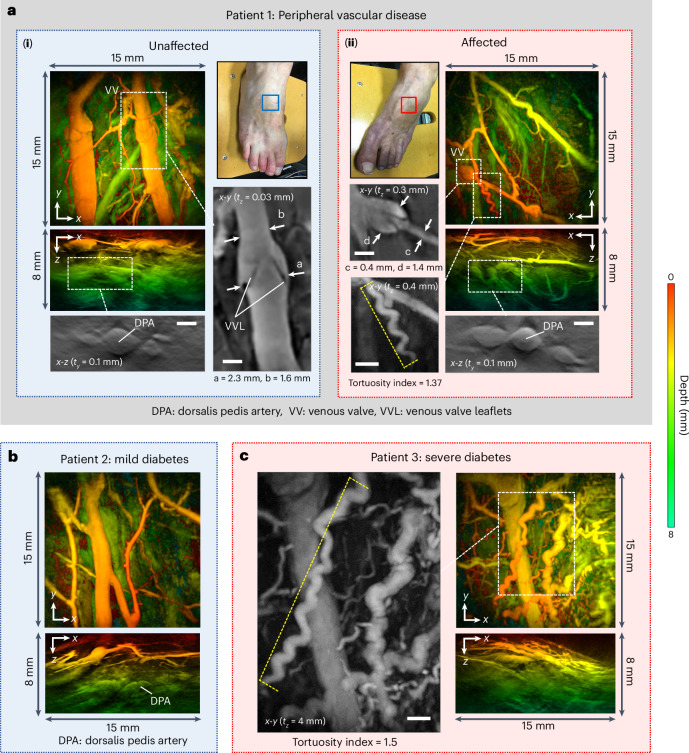
PAT images of feet of patients with suspected peripheral vascular disease. **a**, Patient 1: (**i**) Unaffected right foot. Left: *x*-*y* and *x*-*z* depth-to-colour encoded MIPs. Expanded view greyscale MIPs: Bottom: *x*-*z* MIP showing dorsalis pedis artery (DPA). Right: *x*-*y* MIP showing venous valve. (**ii**) Affected left foot. Right: *x*-*y* and *x*-*z* depth-to-colour encoded MIPs. Expanded view greyscale MIPs showing venous valve (left) and corkscrew vessel (bottom), tortuosity index = 1.37 (measured over length of vessel indicated by dashed yellow line). Bottom right: greyscale *x*-*z* MIP showing DPA. **b**, Patient 2: mild type 2 diabetes, *x*-*y* and *x*-*z* depth-to-colour encoded MIPs. **c**, Patient 3: severe type 2 diabetes. Right: *x*-*y* and *x*-*z* depth-to-colour encoded MIPs. Left: expanded view greyscale MIP showing disorganized irregular vasculature and corkscrew vessel with tortuosity index = 1.5 (measured over length of vessel indicated by dashed yellow line). Scale bars, 1 mm. *t*_*x*_ and *t*_*y*_: slice thicknesses of *y*-*z* and *x*-*z* greyscale MIPs, respectively. Imaging parameters: *λ* = 850, d*x* = d*y* = 108 µm, d*t* = 16.67 ns, PRF = 100 Hz, *N* = 16, A-line rate: 1,400 A-lines per second, scan time *T* = 15 s.

Peripheral vascular disease (PVD) affects more than 25 million individuals across the USA and Europe^64^ and is a complication of diseases such as diabetes that can lead to compromised perfusion resulting in pain, tissue damage and, in severe cases, necessitates limb amputation. While larger vessels can be visualized using conventional duplex ultrasound or magnetic resonance imaging (MRI), the small-vessel changes implicated in PVD that can contribute to adverse consequences such as poor wound healing and amputation cannot be visualized in sufficient detail. Hence, there is a need for sensitive label-free imaging techniques that can be used to detect nascent microcirculatory changes and prompt the early intervention required to prevent the onset of tissue damage. It has been suggested that PAT could play a role in this context^17,18^. To illustrate the potential utility of the scanner in PVD, the feet of patients at risk of small-vessel PVD were imaged in the vicinity of the dorsalis pedis artery (DPA). Following clinical examination, the microcirculation in the right foot of a patient (denoted Patient 1) was assessed as normal, whereas the left foot was symptomatic of impaired perfusion. The PAT image of the unaffected right foot of this patient (Fig. 7a(i)) appears broadly consistent with the images acquired in disease-free volunteers. The affected left foot (Fig. 7a(ii)), however, exhibits differences with evidence of vessel tortuosity which has been linked to microvasculopathy associated with PVD^65^. Figure 7b,c show PAT images of two patients with type 2 diabetes, which is commonly associated with PVD. Patient 2 was diagnosed with mild disease and exhibits an apparently normal vascular morphology. The PAT image of Patient 3, who was diagnosed with more severe disease, reveals a more disorganized, irregular microvascular architecture with several tortuous vessels, some exhibiting a corkscrew structure (Fig. 7c), which in retinal vessels has been linked to diabetes^66,67^. The ability to visualize the lower limb microvasculature in detail as shown in Fig. 7 could be used to study small-vessel PVD linked to diseases such as diabetes with a view to identifying structural microvascular biomarkers that can be used to inform diagnosis and treatment decision-making.

#### Inflammation

**Fig. 8:**
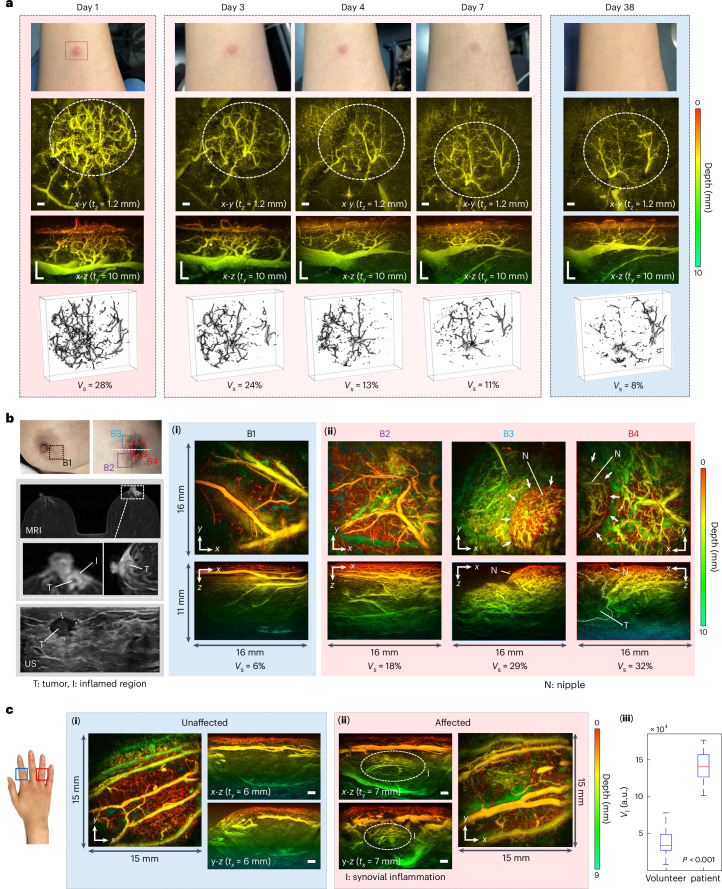
PAT images of inflammatory responses. **a**, Inflammation around raised skin papule over 38 days. *x*-*y* depth-to-colour encoded MIPs (1.2 mm slice thickness; *z* = 0.5–1.7 mm) and *x*-*z* MIPs. The inflamed regions are indicated by the dashed white ellipses. Photograph in blue panel is representative of imaged region at day 38 but was taken non-contemporaneously at a later date. Lower row of images shows 3D skeleton representations of vascular architecture used to estimate vascular density, *V*_s_. Scale bars, 1 mm. *t*_*z*_ and *t*_*y*_: slice thicknesses of *x*-*y* and *x*-*z* greyscale MIPs, respectively. Imaging parameters: *λ* = 850, d*x* = d*y* = 108 µm, d*t* = 16.67 ns, PRF = 100 Hz, *N* = 64, A-line rate: 3,723 A-lines per second, scan time *T* = 5.5 s. **b**, Inflammation in nipple areolar complex (NAC) region of patient with tumour in right breast. Left: photographs, MRI and ultrasound images. MRI images; full view (top), expanded view (middle) around NAC region of affected right breast showing inflamed region (I) and tumour (T). Bottom: ultrasound image showing tumour. Right: (**i**) PAT image (*x*-*y* and *x*-*z* depth-to-colour encoded MIPs) of region B1 on unaffected breast showing low microvascular density, *V*_s_ = 6%. (**ii**) B2, B3 and B4 are locations on the affected breast showing increased vascular densities, *V*_s_ = 18, 29 and 32%, respectively. White arrows delineate nipple (N). Dotted white line on B4 *x*-*z* MIP image represents presumed tumour boundary; see Extended Data Fig. 3 for additional tumour visualization. Imaging parameters: *λ* = 850, d*x* = d*y* = 108 µm, d*t* = 16.67 ns, PRF = 100 Hz, *N* = 16, A-line rate: 1,400 A-lines per second, scan time *T* = 15 s. **c**, Inflammation in synovial region of finger joints in patient with rheumatoid arthritis. (**i**) Unaffected proximal interphalangeal joint on index finger. *x*-*y*, *x*-*z* and *y*-*z* depth-to-colour encoded MIPs. (**ii**) Affected proximal interphalangeal joint on ring finger. *x*-*y*, *x*-*z* and *y*-*z* depth-to-colour encoded MIPs. *x*-*z* and and *y*-*z* MIPs show increased vascular contrast in inflamed synovial region (indicated by dashed white ellipses). Right: box-and-whisker plot showing differences in mean vascular signal *V*_I_ for the volunteer control group (44 joints from 5 healthy volunteers) and the patient group with active disease (17 disease-active joints from 7 patients) joints. For the boxplots, the centre line represents the median, the box limits the first and third quartiles, the whiskers (minima and maxima) 1.5× the interquartile range. *t*_*x*_ and *t*_*y*_: slice thicknesses of *y*-*z* and *x*-*z* greyscale MIPs, respectively. Imaging parameters: *λ* = 850, d*x* = d*y* = 108 µm, d*t* = 16.67 ns, PRF = 100 Hz, *N* = 16, A-line rate: 1,400 A-lines per second, scan time *T* = 15 s. All scale bars, 1 mm.

Visualizing angiogenesis induced by inflammation is relevant to the assessment of a variety of disease and injury processes, among them dermatological conditions, cancer and rheumatoid arthritis. To illustrate the potential application of the scanner in this context, it was used to image three examples of inflammation-driven neovascularization. Figure 8a shows the first of these. Images were acquired longitudinally at 5 different time points of a region of superficial skin inflammation around a raised papule on the forearm following a suspected insect bite. Images were acquired at different times over a period of 7 days during which the papule was judged to be visible by eye. An additional image was acquired 38 days later when the inflammation had fully subsided. The image on day 1 was acquired when the inflammation was judged to be at its apogee. It reveals a dense, chaotic microvascular architecture in the region of the papule that extends from the epidermis to the hypodermis to a depth of ~4 mm. Visual inspection of the sequence of images in Fig. 8a shows the number of vessels in this region gradually diminishing to the presumed baseline over time. This is evidenced quantitatively by the reduction in the vascular density *V*_s_ of the inflamed region from 28% to 8%, where *V*_s_ is the percentage of the image volume occupied by resolvable vessels (see Methods). These results illustrate how the scanner can visualize and quantify changes in the microvascular architecture of the skin over time, attributes that are relevant to the clinical assessment of inflammatory skin disorders such as eczema or dermatitis^20^.

**Extended Data Fig. 3:**
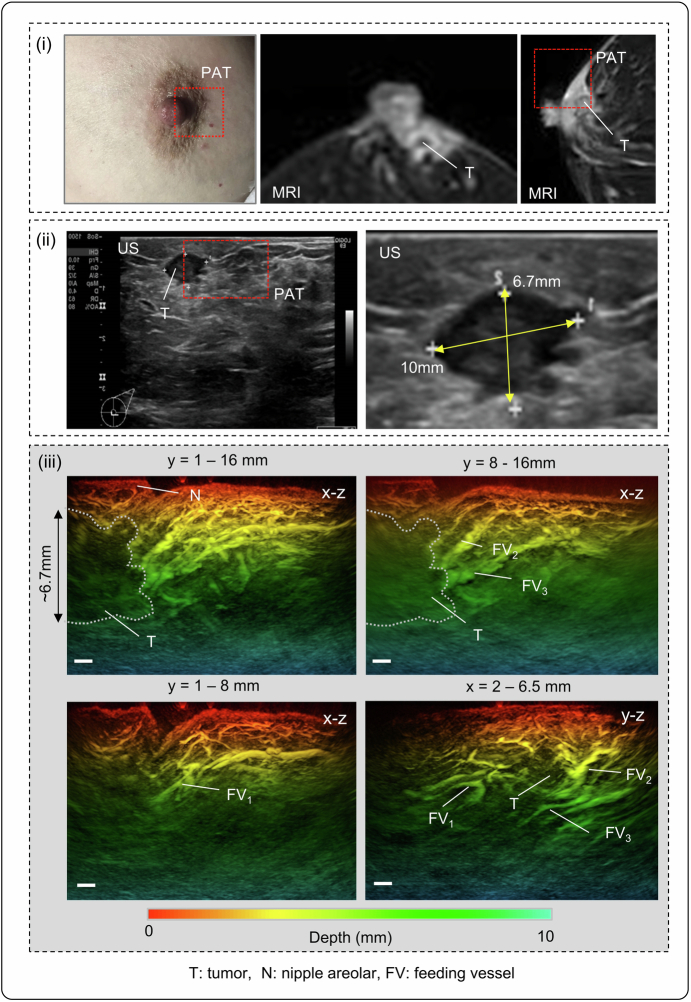
Images of right breast with tumour. (i) Photograph, MRI and (ii) ultrasound (US) images of tumour and approximate region of PAT scan (red dotted box. (iii) PAT images of different slice thicknesses showing presumed tumour (T) boundary and feeding vessels (FV). The tumour region exhibits low photoacoustic contrast suggesting lower vascular density than surrounding tissue. Imaging parameters: λ=850, dx=dy=108µm, dt=16.67ns, PRF=100Hz, N=16, A-line rate: 1400 A lines/s, scan-time T=15s.

In the second example, the scanner was used to visualize the skin inflammation linked to breast cancer. This can arise when a tumour provokes an inflammatory response that produces skin neovascularization, for example, in the nipple areolar complex (NAC) region. In patients with locally advanced disease of this nature, even if the tumour is beyond the penetration depth range of the scanner, the neovascularization can be relatively superficial (<5 mm) and thus can be visualized. This is illustrated in Fig. 8b. Figure 8b(ii) shows PAT images acquired in different areas (B2, B3, B4) of the NAC region in a patient with a tumour in the right breast, identified via diagnostic ultrasound and MRI. Compared with the PAT image of the unaffected left breast (Fig. 8b(i)), the images of the right breast reveal an increase in contrast due to skin neovascularization, also evident in the MR images but not via ultrasound examination or visual inspection. Image contrast in the inflamed region was quantified by calculating *V*_s_ as described above, with the ROI selected to exclude the skin and nipple. For the unaffected left breast, *V*_s_ = 6%, whereas the mean *V*_s_ of the three images of the inflamed region in the right breast was 26%. Although the objective was not to visualize the tumour, its boundary is discernible in the *x*-*z* MIP for position B4; Extended Data Fig. 3 provides additional visualizations of this region showing the tumour and its supplying vasculature. The ability to visualize inflammatory responses in this way could find application, for example, in the study of the aetiology of tumour-driven inflammation in cancer.

In the third example, the scanner was used to image the joints of patients with rheumatoid arthritis. This is a disease of the joints characterized by inflammation of the synovial membrane, which affects >20 million people worldwide^68^, causing discomfort, immobility and, if left untreated, permanent loss of joint use. The inflammation produces neovascularization that could potentially serve as a marker of disease severity but is inadequately visualized by existing imaging modalities such as conventional B-mode and Doppler ultrasound. This has led to suggestions that PAT could play a role in the clinical assessment of rheumatoid arthritis^21–23^. To illustrate the potential application of the scanner in this context, images of suspected inflamed hand joints were acquired.

Figure 8c shows examples of PAT images of the interphalangeal joints in a patient with a diagnosis of rheumatoid arthritis in the right hand; the diagnosis was based on the observation of synovial thickening and increased blood flow observed under clinical Duplex ultrasound examination, a biochemical inflammatory marker and patient-reported symptoms. Consider the vertical *x*-*z* and *y*-*z* MIPs in Fig. 8c. Compared with the image of the unaffected joint in the left hand of the same patient (Fig. 8c(i)), the affected joint in the tendon region (indicated by the white dotted ellipse in Fig. 8c(ii)) exhibits increased contrast. Given the more subtle nature of the contrast compared with that evident in Fig. 8a,b, this preliminary observation was consolidated by undertaking a more extensive study. This involved scanning 17 joints of a further 7 patients with high disease activity (>5.1 Disease Activity Score-28 score) and 44 joints of a control group of 5 healthy volunteers below the age of 30 yr. The vascular contrast in the synovial region was then quantified by calculating the number of voxels *V*_I_ above the background (see Methods). This approach was used instead of the vessel-by-vessel-based analysis used in the examples shown in Fig. 8a,b to estimate *V*_s_. This is because joint inflammation was observed to manifest itself over time as an increase in subresolution spatially averaged vascular contrast, rather than a proliferation of clearly resolved individual vessels. The results are summarized in the box-and-whisker plot in Fig. 8c(iii), which show a significant difference (*P* < 0.001) between the patient group with active disease (median *V*_I_ = 141,314) and the volunteer control group (mean *V*_I_ = 39,511). This suggests that even subtle subresolution inflammation-driven neoangiogenesis can be detected, as well as that associated with the clearly resolvable changes in microvascular morphology observed in Fig. 8a,b.

## Discussion

A high-resolution 3D photoacoustic scanner based on the FP ultrasound sensor concept has been developed and evaluated to assess its potential as a tool for clinical vascular imaging. The key engineering advance that has been made relates to acquisition speed. Early-generation FP scanners^43^ provided high 3D image quality but at the cost of long scan-times, thus precluding practical clinical use. This limitation has been overcome by variously parallelizing the FP sensor readout, operating at high excitation laser PRFs and employing compressed sensing. High-quality 3D images can now be acquired without compromising image quality in a few seconds rather than the several minutes of early scanners. Even shorter scan-times of a few hundred ms are achievable if modest reductions in image fidelity or lateral FOV can be accepted. The significant increase in acquisition speed that has been achieved has advanced the clinical applicability of the technology. It now enables high-quality in vivo images to be repeatably acquired without appreciable motion-related artefacts, permits real-time image display during probe positioning and allows the visualization of time-varying perfusion and other haemodynamic events. Moreover, the scanner has been shown to be a reliable and versatile instrument that can provide high-fidelity in vivo images at a diversity of anatomical sites with comparable patient acceptability and convenience to a conventional clinical ultrasound scanner. All of these sets the scene for the clinical translation of the technology as a tool for the assessment of diseases associated with microvascular changes.

### Clinical applications

This study has shown that the scanner can provide rapid, highly detailed volumetric images of vascular anatomy and function. This is evidenced by the imaging studies on healthy volunteers. These studies demonstrated that high-resolution 3D images to depths approaching 15 mm can be acquired, revealing capillary loops, venules, arterioles and large mm-scale arteries and veins, as well as other structures such as venous valves, skin sulci and hair follicles. In addition, the clinical case studies showed that the geometrical parameters of the superficial vasculature could be quantified and followed longitudinally over time. Moreover, they illustrated how vascular abnormalities such as increased vessel tortuosity which has previously been linked to PVD, and the neovascularization associated with inflammation can be visualized and quantified. Altogether, these results suggest that there is a variety of structural vascular characteristics that can be visualized using the system, which could potentially inform the detection, diagnosis and treatment monitoring of disease and injury processes characterized by microcirculatory abnormalities.

In cardiovascular medicine, the scanner could be used to provide early detection of the skin microvascular changes associated with PVD that precede tissue damage in the lower limb in patients with diabetes. This could provide more effective monitoring for planning angioplasty and other therapeutic interventions before tissue death and ulceration occurs. As shown in the current study, the technology also lends itself to the assessment of inflammatory conditions. In patients with rheumatoid arthritis, it could be used to assess synovial inflammation to identify which patients require treatment and monitor them over time to ensure optimal dosing to minimize damage and loss of joint mobility. In a similar fashion, it could be used for the assessment of inflammatory skin conditions such as eczema or dermatitis and inflammation associated with cancer, infection and superficial soft-tissue injury due to burns or wounds. A further promising area of application is surgical guidance^69^. Reconstructive procedures such as flap surgery require detailed knowledge of the vasculature in and around the transplanted tissue. The scanner could therefore be used preoperatively to image and assess perforators to help identify the optimum donor tissue site, intraoperatively to guide flap positioning to ensure adequate connection between the donor and acceptor vascular networks and post-operatively to monitor perfusion and guide recovery. Similarly, it could be used in cancer surgery to delineate the margins of vascularized tumours in the skin or oral cavity and help plan their surgical excision. In open or laparoscopic surgery, endoscopic implementations^70–72^ could be used intraoperatively to guide the treatment of tumours in the liver and other abdominal organs or surgical procedures in the GI tract.

### Future technical developments

The FP sensor technology is versatile and flexible with considerable scope to adjust acquisition speed, resolution, penetration depth, functionality and form factor. In this sense, it can be regarded as a generic technology around which a range of imaging instruments, each tailored to meet the requirements of specific applications, could be developed.

For example, scanners operating at higher frame rates than demonstrated in the current study could be realized for applications that require visualizing dynamic vascular biomarkers. Increasing the number of interrogation beams by a factor of 2–3 would provide a commensurate increase in frame rate to ~5 fps without compromising image quality. Although further parallelization with the current multibeam readout architecture is likely to be prohibitive in terms of technical complexity and cost, alternative full-field massively parallelized camera-based readout schemes^73,74^ have the potential to overcome this and achieve higher frame rates. Increasing the frame rate by operating at high (kHz) PRFs is also possible; laser diode arrays providing kHz PRFs with mJ scale pulse energies at shorter, more deeply penetrating wavelengths than the 1,064 nm wavelength of the 1 kHz PRF fibre laser used in the current study are available commercially. Inevitably however, increasing the PRF incurs an SNR cost due to the need to limit the pulse energy to comply with safe laser exposure limits. Subsampled acquisition provides an opportunity to address this. As shown in Fig. 5b, high-quality images can be reconstructed with even highly subsampled data, albeit at the cost of long image reconstruction computation times although there is the potential to reduce this using learned reconstruction methods^60^. Taking all of the above into account, a 3D frame rate of 10 fps should be achievable without significantly compromising image quality via a combination of a factor of 2 increase in parallelization and a modest subsampling of a factor of 2 or 3. For applications that can tolerate a reduction in image SNR, a combination of ×10 subsampling and a 1 kHz PRF could enable 3D frame rates in excess of 100 fps to be achieved.

In scenarios where real-time 3D imaging is required, the main computational challenge is to achieve sufficiently short latencies, that is, the time span between data acquisition and image visualization, which is dominated by the image reconstruction time. For fully sampled acquisition, the latency of our CPU-based implementation is between 0.48–0.8 s on a desktop PC. Optimized GPU-based implementations or parallelization over multiple computing nodes with the same hardware could decrease the latency substantially. In addition, real-time visualization of high-resolution 3D images is not required for some tasks, for example, to assist probe placement. This offers the opportunity to reduce latencies by employing pseudo-3D backprojection reconstruction methods, a concept developed for computed tomography (CT) in which selected image volumes or planes are computed directly, bypassing the need for a computationally burdensome full 3D reconstruction.

Penetration depth and spatial resolution can be adjusted by modifying the FP sensor design. It has been shown that, by increasing the sensor finesse through the use of a plano-concave cavity geometry^32,75^, detection sensitivity can be increased by at least an order of magnitude, offering the prospect of increasing imaging depth to 2–3 cm. The acoustic bandwidth can readily be increased to 100 MHz by reducing the FP polymer film thickness to achieve higher spatial resolution for ultra-high-resolution vascular imaging applications, for example, visualizing the highly superficial dermal vasculature at capillary level.

Other imaging modalities can readily be incorporated to provide complementary anatomical or physiological information. The transparent nature of the sensor allows straightforward integration of pure optical imaging techniques such as optical coherence tomography (OCT)^76^ or fluorescence imaging. A 3D ultrasound imaging capability^77^ could be implemented by using a dichroic absorptive coating deposited on the sensor. This would enable a dual-mode imaging modality in which the ultrasound image provides morphological mechanical contrast complementary to the PAT vascular contrast. As well as visualizing anatomical features indistinguishable with PAT, it would aid clinical translation by providing an anatomical imaging landscape recognizable to clinicians familiar with ultrasound that helps in the interpretation of the PAT image.

Finally, the concept lends itself to a multitude of form factors. The large size and weight of the current galvanometer-based probe head makes it somewhat cumbersome for routine clinical use. By replacing the galvanometer scanners with a compact micro-electromechanical systems (MEMs) based scanner^72^, a hand-held probe head of similar dimensions, ergonomic shape and weight to a conventional clinical ultrasound probe could be realized. Moreover, there is scope to develop miniature endoscopic or intracavitary probes by interrogating the FP sensor via an optical fibre bundle^70,71^. Not least, by virtue of the all-vacuum deposition fabrication techniques used to manufacture the sensor which enables batch fabrication at low unit cost, the technology lends itself to minimally invasive applications where single-use disposable sensors are required.

In summary, we have reported a high-fidelity 3D PAT scanner that can provide rapid, detailed in vivo 3D images of superficial vascular anatomy in clinically acceptable acquisition times. The level of image detail that it provides suggests that it could find application as a tool for the clinical detection, diagnosis and treatment monitoring of diseases such as diabetes or cancer that are characterized by microcirculatory abnormalities. The demonstrated combination of high image fidelity, fast acquisition, design versatility and the practical nature of the technology sets the scene for its clinical translation in oncology, cardiovascular medicine, dermatology, image guided surgery and other medical specialties.

## Methods

### Fabry–Perot ultrasound sensor

The FPI spacer was formed by the vacuum deposition of Parylene C^78^. The mirrors of the FPI comprised a dichroic dielectric stack transparent between 560 nm and 1,300 nm and highly reflective between 1,460 nm and 1,630 nm (see Supplementary Fig. 1). A Parylene C layer a few microns thick was deposited over the FPI to protect it from water ingress and mechanical damage. Two different sensors of similar design were used: for the volunteer studies, the sensor spacer thickness was 26.6 µm, providing an estimated -3 dB bandwidth of 31.5 MHz. For the clinical case studies, the sensor spacer thickness was 29.6 µm providing an estimated -3 dB acoustic bandwidth of 27.6 MHz. The effective signal bandwidth was the same irrespective of the sensor used since a digital 50 kHz–20 MHz bandpass filter was applied to the detected photoacoustic waveforms in all cases, except for the spatial resolution measurements and high-resolution scan-mode images (Fig. 4) for which the filter bandwidth was 50 kHz–30 MHz. For the 26.6 µm sensor, the reflectivity finesse was *F* = 76.6, the fringe visibility *V* = 0.78 and the free spectral range FSR = 26.6 nm. For the 29.6 µm sensor, *F* = 33.8, *V* = 1.00 and FSR = 23.6 nm.

### Scanner hardware

The interrogation laser system comprised a Santec external cavity laser, tunable between 1,500 nm and 1,630 nm and amplified by a pair of C-band erbium-doped fibre amplifiers. To record the output of the FP sensor, custom-designed InGaAs photodiode-transimpedance units with AC and DC-coupled outputs were used; the bandwidth of the AC-coupled output was 50 kHz–75 MHz. Each photodiode unit was connected to an input of a multichannel RF acquisition system of up to 64 channels with 60 Ms s^−1^ (megasamples per second) sampling rate. The interrogation beams were scanned using a conjugate galvanometer-based scanner (Supplementary Fig. 2). The maximum scanning angle used was 15.2°, the focused interrogation beam spot diameter (1/e^2^) was 49 µm and the Rayleigh range 2.47 mm. The system crosstalk was evaluated by delivering light to one of the optical fibres close to the centre of the 64-fibre bundle at the same optical power that was used when imaging while simultaneously monitoring the photodiode signals of the remaining 63 channels. On the channels immediately adjacent to the illuminated one, the crosstalk was 0.5%. On all other channels, no crosstalk could be detected.

Three excitation laser systems were variously used depending on the PRF requirements. Two were Type II 532 nm pumped OPO-based laser systems. One was an Ekspla Photosonus-X operating at 100 Hz (depicted in Fig. 1b) and the other was an Innolas EVO 200 that could operate at either 100 Hz or 200 Hz. Both provided pulse energies in excess of 10 mJ, pulse durations of 5 ns and a signal wavelength range between 660 nm and 1,300 nm. The outputs of both systems were coupled into 1.5-mm-diameter optical fibres. The third excitation laser was a large-core Yb pulsed fibre laser based on a master oscillator power amplifier architecture^49^ custom designed and built by the Optoelectronics Research Centre, Southampton University. It provided a 1,064 nm output, a pulse energy of 10 mJ, adjustable pulse duration (10–500 ns) and PRFs of 100–1 kHz. In this study, the pulse energy and duration used were 2.7 mJ and 20 ns, respectively.

### System characterization

The system was characterized using similar methods to those described in ref. ^37^. The NEP distribution shown in Fig. 1e was obtained by measuring the FP sensor signal at 34,560 scan points on the sensor in response to a plane wave of known peak pressure emitted by a calibrated planar 3.5 MHz piezoelectric transducer^37^. At each scan point, the signal amplitude and root mean square noise (over a 20 MHz measurement bandwidth) were measured to estimate the NEP. To obtain the NEP distribution for a single-beam scanner for comparison, light from the interrogation laser was coupled into only a single fibre within the 64-beam array. NEP measurements were then acquired at 34,560 scan points, with the bias wavelength optimally set at each scan position.

The sensor frequency response (Fig. 1c) was obtained using a substitution method based on a broadband laser-generated ultrasound source and a calibrated reference detector of known frequency response^37^. The in vivo acoustic frequency tissue spectrum shown in Fig. 1c (dashed line) was obtained by averaging the frequency spectra of 34,560 A-lines acquired in a scan of the human palm. The spatial resolution as a function of position was estimated by scanning over 21 × 9.5 mm^2^ a grid of plastic line absorbers immersed in Intralipid and reconstructing images of the phantom for three scan step sizes: d*x* = d*y* = 54 µm, d*x* = d*y* = 108 µm and d*x* = d*y* = 162 µm (Supplementary Figs. 3 and 4). To estimate the lateral and vertical resolution, the edge spread and line spread functions respectively were measured^37^.

### Signal processing, image reconstruction and visualization

Before image reconstruction, the raw photoacoustic waveforms were preprocessed by applying a bandpass filter; 50 kHz–20 MHz was used in most cases. For the high-resolution scan mode and spatial resolution measurements, the upper cut-off frequency was increased to 30 MHz. For the image in Extended Data Fig. 2, a 2–20 MHz filter was used. The recorded pressure time-series data were spatially interpolated onto a ×2 finer grid, except for the dynamic imaging examples (Fig. 6) for which a factor of ×3 interpolation was used^79^. The tissue sound speed was then estimated using an autofocus method^80^ and input to the reconstruction algorithm. All images, except the subsampled images in Fig. 5b, were reconstructed using a *k*-space backprojection algorithm^51^. The reconstructed image was then interpolated onto a ×2 finer grid. The image reconstruction was implemented using *k*-Wave, an open-source toolbox developed at UCL for the time-domain simulation and reconstruction of photoacoustic and ultrasound wave fields (www.k-wave.org; ref. ^81^).

By employing a fast C++ CPU optimized computational implementation, image reconstruction time has been reduced by a factor of 5 over a previous MATLAB implementation^81^. Reconstruction time depends on scan area, spatial sampling interval and the number of points in the time record. For example, for a scan area of 21 × 19.5 mm^2^, d*x* = d*y* = 108 µm and a record length of 600 time points, the reconstruction time was 0.8 s, decreasing to 0.48 s for a 15 × 15 mm^2^ scan for the same spatial-temporal sampling intervals using a desktop PC.

The subsampled images in Fig. 5b were reconstructed using an iterative model-based reconstruction algorithm based on least squares minimization with a total variation regularization term and a non-negativity constraint, described further in Supplementary Note 3 and ref. ^50^. Regularization parameters were chosen to (1) suppress the most visible reconstruction artefacts, (2) retain most visible small vessels and (3) correct the most obvious geometrical distortion of clearly visualized vessels. The regularization parameter for the fully sampled image was *λ* = 12 × 10^−4^. The regularization parameter for each subsampled image^50^ was *λ* multiplied by the respective subsampling factor. A maximum of 50 iterations were computed for each example. Computing the reconstruction of the images in Fig. 5b (192 × 180 × 396 voxels) took 17, 22, 23 and 25 min for 100, 50, 25 and 12.5% subsampling factors, respectively, by using optimized CUDA code on a Supermicro SuperServer SYS-420GP-TNR: it has 2 × 16-core Intel Xeon Silver 4314 2.40 GHz CPUs (Ice Lake), 1,024 GB of 3,200 MHz DDR4 memory, and 3× NVIDIA Tesla A100 Ampere GPUs each with 6,912 CUDA cores and 80 GB of memory.

Since the excitation beam diameter was larger than the scan area, in some cases, images were reconstructed slightly outside the scan footprint to increase the lateral FOV; for example, the images in Fig. 2c,d were reconstructed over a 22 × 22 mm area although the physical scan area was 21 × 19.5 mm^2^. To aid the visualization of deeper-lying features, a first-order correction for optical and acoustic attenuation was used^79^. This was implemented by scaling the image intensity in the depth direction with an exponential function, the exponent of which was in the range 100–150 m^−1^. All MIPs are displayed using a linear image intensity scale. The intensity scale used for all greyscale images was linear from black (0) to white (1).

Unless otherwise indicated, all colour-to-depth encoded MIPs are full-thickness projections, whereas greyscale MIPs are reduced-thickness projections; *t*_x_ and *t*_y_ denote the *x* and *y* slice thicknesses, respectively, in these MIPs.

### Image quantitation

Image CNR was estimated as follows. To determine the contrast, the mean pixel intensity at each *x*-*y* plane was first computed to obtain a measure of the background. The background was then subtracted from all pixels throughout the image volume and the peak value of the mean intensity as a function of depth taken to represent the contrast. For the images in Fig. 5a, the contrast was determined within the volume demarcated by the dashed white rectangles as these define the common anatomical ROI for each case. In all other cases, the entire image volume was used to estimate the contrast. The noise was determined by selecting a volume devoid of image features and calculating the root mean square value of the pixel intensities within it.

To quantify vascular contrast, two metrics were used. When individual vessels could be resolved (as in Fig. 8a,b), the vascular density *V*_s_ which represents the percentage of the image volume occupied by resolvable vessels was used. *V*_s_ was determined by segmenting the vasculature, forming a 3D skeleton visualization and estimating the number of non-zero pixels as a percentage of the total number of pixels in the image. The segmentation of the vasculature was achieved using *K*-means clustering^82^, implemented with the MATLAB function ‘imsegkmeans3’. This approach classifies image pixels on the basis of intensity by forming clusters in intensity space where pixel intensities within each cluster are maximally close to each other and distant from pixel intensities in other clusters. This provided effective segmentation of the resolvable vasculature without the need to define a threshold. To quantify subresolution vascular contrast (as in Fig. 8c), the number of voxels above the background within the volume of interest was estimated and denoted *V*_I_. The tortuosity index (Fig. 7) is given by the ratio of the vessel length to the straight-line distance between two points along the vessel^67^.

### Volunteer and patient studies

Volunteers were recruited from the Department of Medical Physics and Biomedical Engineering at UCL with ethical permission granted by University College London Research Ethics Committee (Project ID: 1133/001). Patients were recruited from relevant clinics at UCL Hospitals NHS Trust under NHS Research Ethics Committee approval (IRAS Project ID number: 206196). Written informed consent was obtained from all participants.

### Reporting summary

Further information on research design is available in the Nature Portfolio Reporting Summary linked to this article.

## Supplementary information

Main Supplementary Information Supplementary figures, notes and video captions.

Reporting Summary

Supplementary Video 1 2D dynamic PAT imaging of the wrist region at 33 fps (offline reconstruction).

Supplementary Video 2 2D dynamic PAT imaging of radial artery at 33 fps (offline reconstruction).

Supplementary Video 3 2D dynamic PAT imaging of wrist region at 25 fps (online reconstruction).

Supplementary Video 4 3D dynamic imaging as probe is moved across fingertip at 16.7 fps (refresh frame rate) using 10% sliding window (offline reconstruction).

Supplementary Video 5 3D dynamic imaging as probe is moved across the palm at 3 fps (refresh frame rate) using 50% sliding window (online reconstruction).

Supplementary Video 6 3D dynamic imaging of perfusion following arterial occlusion at 16.7 fps (refresh frame rate) using 10% sliding window (offline reconstruction).

Supplementary Video 7 2D dynamic multiwavelength imaging.

Supplementary Video 8 Step sequence of in vivo 3D images acquired at 4 different wavelengths and shown in Supplementary Fig. 5.

## Data Availability

The raw image data sets and reconstructed-image data are available from the authors for research purposes on reasonable request.

## Code availability

All custom MATLAB scripts used to process and analyse the data are available from the authors for research purposes on reasonable request.
