# Supplementary material for: A fast all-optical 3D photoacoustic scanner for clinical vascular imaging
Source: Nat Biomed Eng. 2024 Sep 30;9(5):638–55. doi: 10.1038/s41551-024-01247-x (PMC12092260; doi:10.1038/s41551-024-01247-x)
Supplement: Supplementary file 1 — Supplementary figures, notes and video captions. [file 41551_2024_1247_MOESM1_ESM.pdf]

nature biomedical engineering

Supplementary information

https://doi.org/10.1038/s41551-024-01247-x

A fast all-optical 3D photoacoustic scanner for clinical vascular imaging

In the format provided by the authors and unedited

Contents

Supplementary Fig. 1| Simulated transmission spectra of FPI mirror coatings.

Supplementary Fig. 2| Conjugate scanner schematic.

Supplementary Fig. 3| Photoacoustic signal emitted from one of the absorbers in the phantom used to measure the lateral spatial resolution.

Supplementary Fig. 4| Effect of spatial sampling interval on lateral resolution.

Supplementary Fig. 5| Multiwavelength images acquired in the wrist region of a human volunteer at 700nm, 808nm, 850nm and 940nm.

Supplementary Fig. 6| Effect of scan-time T on in vivo images of the palm of a healthy volunteer.

Supplementary Note 1| Fabry Perot ultrasound sensor modelling.

Supplementary Note 2| MPE calculations.

Supplementary Note 3| Iterative model-based image reconstruction approach for compressed sensing data.

Supplementary Fig. 7| Effect of sub-sampling factor (SSF) on CNR, MSE and spatial resolution.

Supplementary Fig. 8| In vivo sub-sampled PAT images reproduced from Fig. 5b in main paper.

Supplementary Fig. 9| Effect of NEP distribution on image quality.

Supplementary Video 1| 2D dynamic PAT imaging of the wrist region at 33 fps (offline reconstruction).

Supplementary Video 2| 2D dynamic PAT imaging of radial artery at 33 fps (offline reconstruction).

Supplementary Video 3| 2D dynamic PAT imaging of wrist region at 25 fps (online reconstruction).

Supplementary Video 4| 3D dynamic imaging as probe is moved across finger-tip at 16.7 fps (refresh frame rate) using 10% sliding window (offline reconstruction).

Supplementary Video 5| 3D dynamic imaging as probe is moved across the palm at 3 fps (refresh frame rate) using 50% sliding window (online reconstruction).

Supplementary Video 6| 3D dynamic imaging of perfusion following arterial occlusion at 16.7 fps (refresh frame rate) using 10% sliding window (offline reconstruction).

Supplementary Video 7| 2D dynamic multiwavelength imaging.

Supplementary Video 8| Step sequence of in vivo 3D Images acquired at 4 different wavelengths shown in Supplementary Fig. 5.

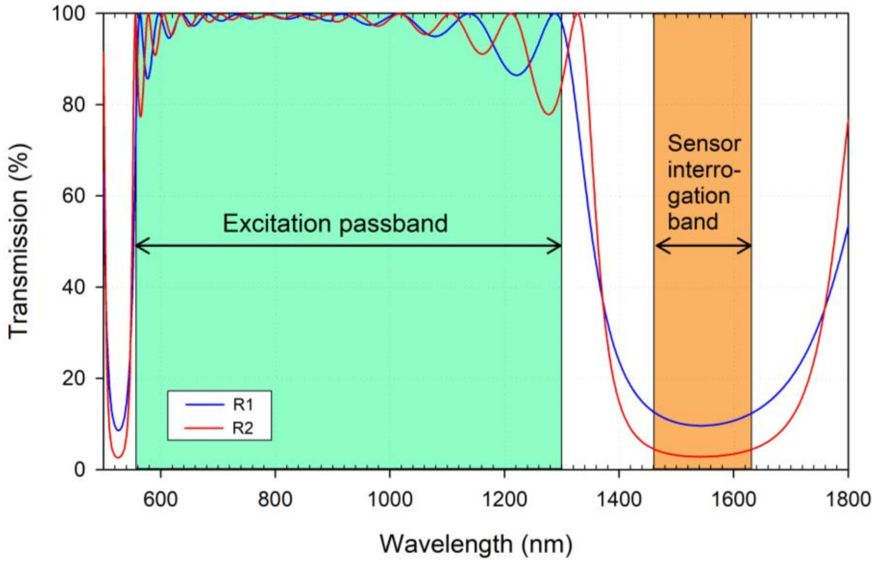

Supplementary Fig. 1| Simulated transmission spectra of FPI mirror coatings showing excitation laser wavelength pass band (560nm – 1300nm) where the mirrors exhibit high transmission and high reflectivity interrogation laser wavelength range (1460nm - 1630nm). R1 is the mirror coating adjacent to the wedged substrate and R2 is the outer mirror coating.

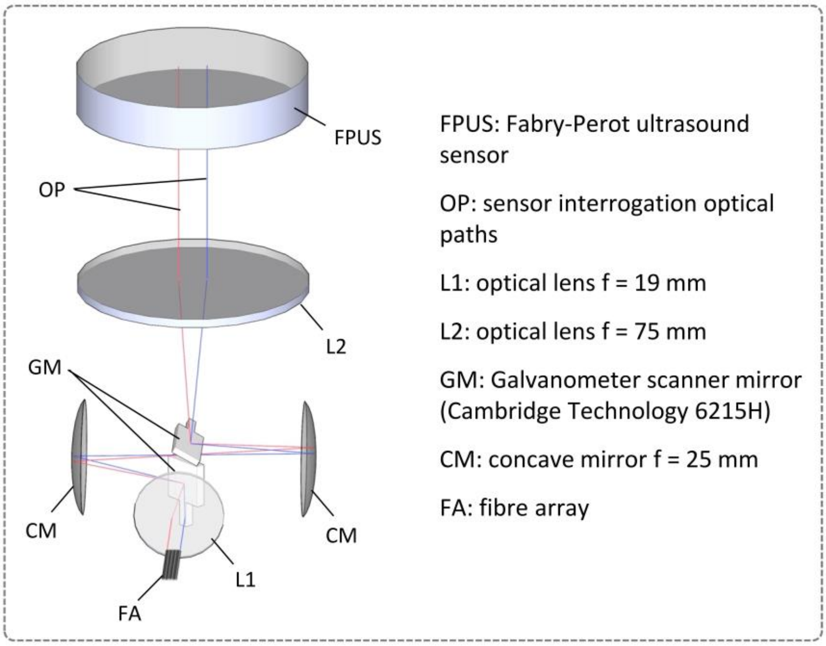

Supplementary Fig. 2| Conjugate scanner schematic

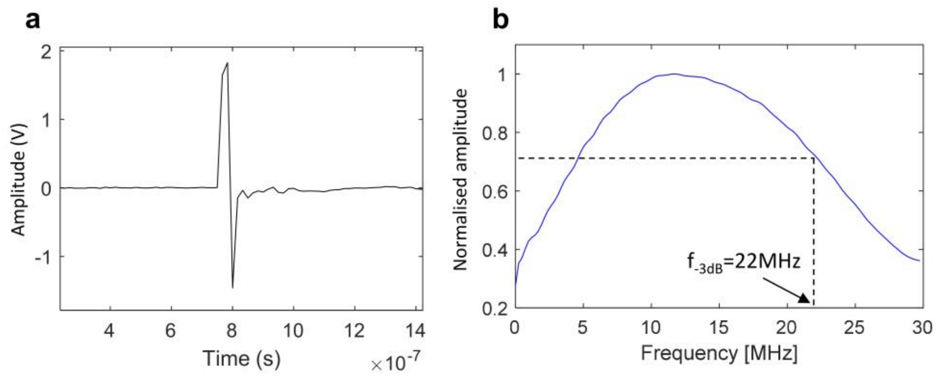

Supplementary Fig. 3| Photoacoustic signal emitted from one of the absorbers in the phantom used to measure the lateral spatial resolution. The absorber was located at the centre of the scan area and directly beneath the detection point at a depth of z=1mm. (a) Detected time-resolved signal and (b) acoustic frequency spectrum.

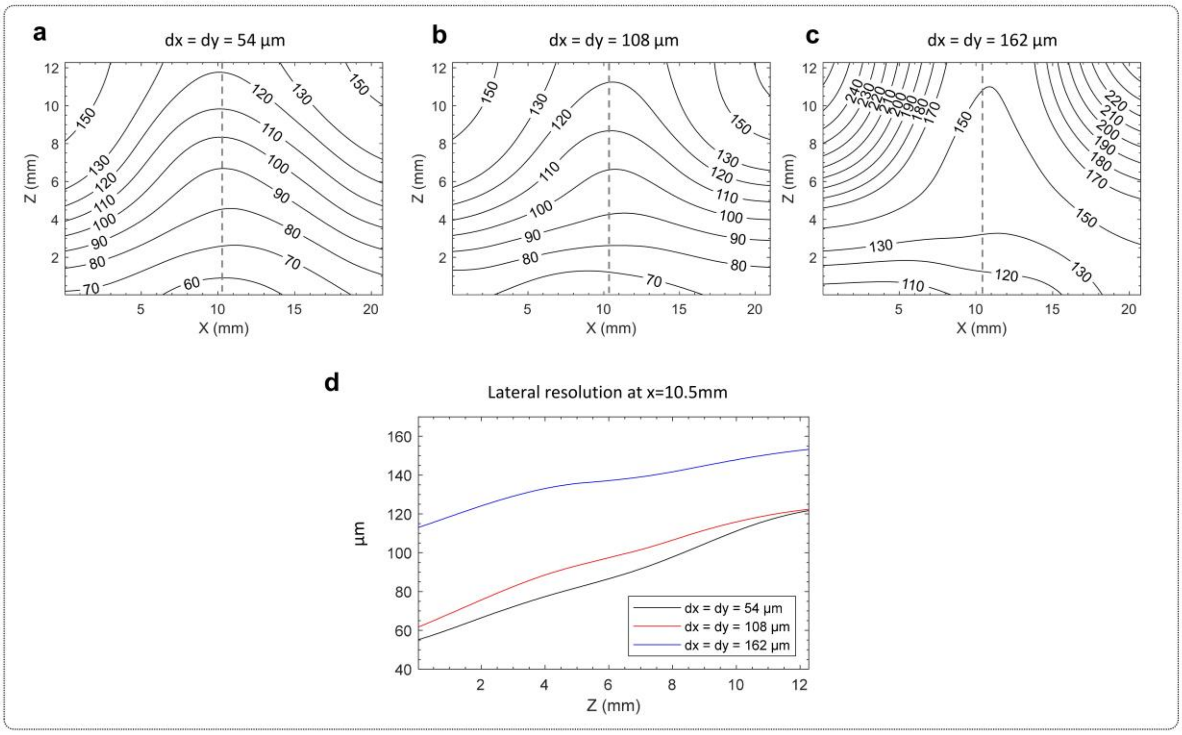

Supplementary Fig. 4| Effect of spatial sampling interval on lateral resolution. (a) dx=dy=54 µm, (b) dx=dy=108 µm, (c) dx=dy=162 µm, (d) Depth dependent lateral resolution at centre of field of view (x=10.5mm) for each sampling interval. The lateral resolution for dx=dy=54 µm is similar to dx=dy=108 µm because the measurement is limited by the source bandwidth (f_{-3dB}=22 MHz, see Supplementary Fig. **3**), rather than the system detection bandwidth. For dx=dy=162 µm, spatial undersampling becomes significant resulting in reduced lateral resolution. Imaging parameters: λ=875nm, dt=16.67ns, PRF=100Hz, N=64, A-line rate: 6400 A lines/s.

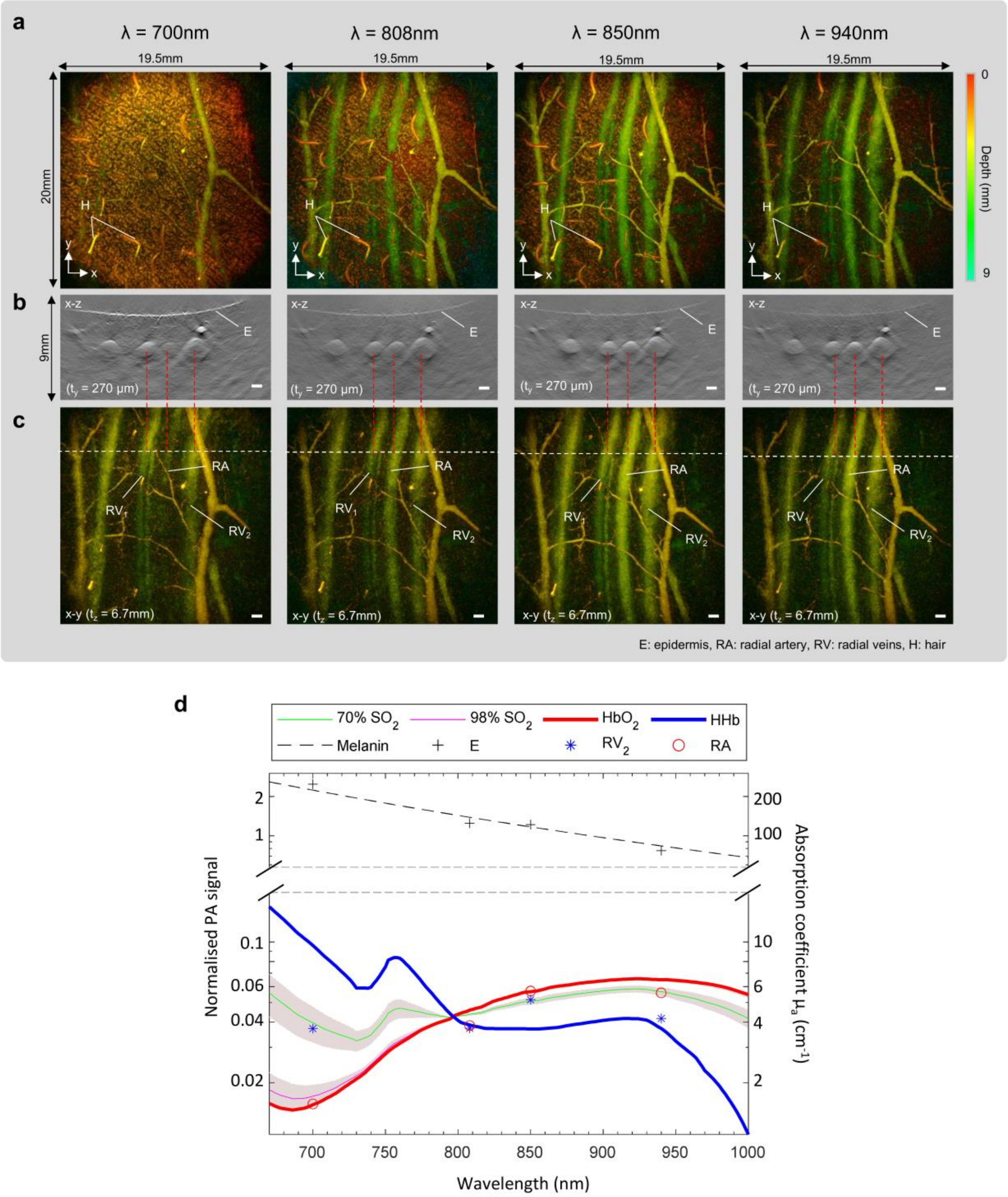

Supplementary Fig. 5| Multiwavelength images acquired in the wrist region of a human volunteer at 700nm, 808nm, 850nm and 940nm (see also Supplementary Videos 7,8). (a) x-y depth-to-colour encoded MIPs from z=0 to 9 mm, (b) greyscale x-z MIPs of slice thickness t_y=270µm, (c) x-y depth-to-colour encoded MIPs but from z=2 mm to 9 in order to remove the skin contrast and enable clearer visualisation of the underlying blood vessels. (d) Literature absorption spectra of melanin[1], oxyhaemoglobin[2], deoxyhaemoglobin[2], arterial (SO_2=98%) and venous blood (SO_2=70%)[3]. The grey shaded regions demarcate the physiological range of arterial and venous blood saturations, 95%–100% and 60%-80% respectively[3]. Overlaid are data points representing the normalised image intensity of regions corresponding to the epidermal layer (E) and the radial artery (RA) and the right hand vein (RV_2) at each wavelength. These data points were obtained from the x-y MIPs (following image registration) by estimating the mean pixel intensity within ROIs corresponding to the skin, RA and RV_2. Imaging parameters: dx=dy=108µm, dt=16.67ns, PRF=100Hz, N=64, A-line rate: 6400 A lines/s, scan-time: T=5.4s per image. All scale bars: 1mm.

From the x-y MIPs in (a) it is apparent that the strong absorption of melanin in the epidermis provides high contrast at 700nm which obscures the underlying blood vessels. As the wavelength

increases, melanin absorption decreases and the blood vessels becomes more visually prominent. From (b) and (c) it is evident that the vascular contrast initially increases with wavelength from 700nm to 808nm as both venous and arterial blood absorption increases. At 940nm, the contrast of the radial artery (RA) and the two radial veins (RV_1 and RV_2, hereon referred to collectively as "the RVs") decreases despite increasing blood absorption, a consequence of the nearby 975nm water absorption peak. The relative contrast of the RA and RVs is also wavelength dependent. This is most obvious in (b) and (c) where it can be seen that at 700nm the RA exhibits significantly reduced contrast compared to the adjacent RVs - indeed the RA is almost invisible at this wavelength due to the x2.3 higher absorption coefficient of venous blood at SO_2=70% compared to arterial blood at SO_2=98%. As the wavelength is increased, the differential contrast between the RA and the RVs decreases to a minimum at 808nm, thereafter increasing at longer wavelengths but with the RA contrast now higher than that of the RV.

Although the data in (d) suggests that the skin and vascular image contrast are broadly consistent with the spectral dependencies of melanin and venous and arterial blood absorption, there are inevitably discrepancies due to the confounding influence of spectral colouring[3,4,5]. For the measurements corresponding to the epidermis (E) this effect is small due to its superficial location. Spectral colouring can be expected to be greater for the more deeply lying blood vessels. However, the alignment of the RA and RV_2 measured data points with the corresponding literature spectra suggests it is still modest for 700mn, 808nm and 850nm although less so at 940nm due to the nearby 975nm water absorption peak. The latter attenuates the light incident on the blood vessels and depresses their measured photoacoustic spectra, an observation consistent with the results in reference 6.

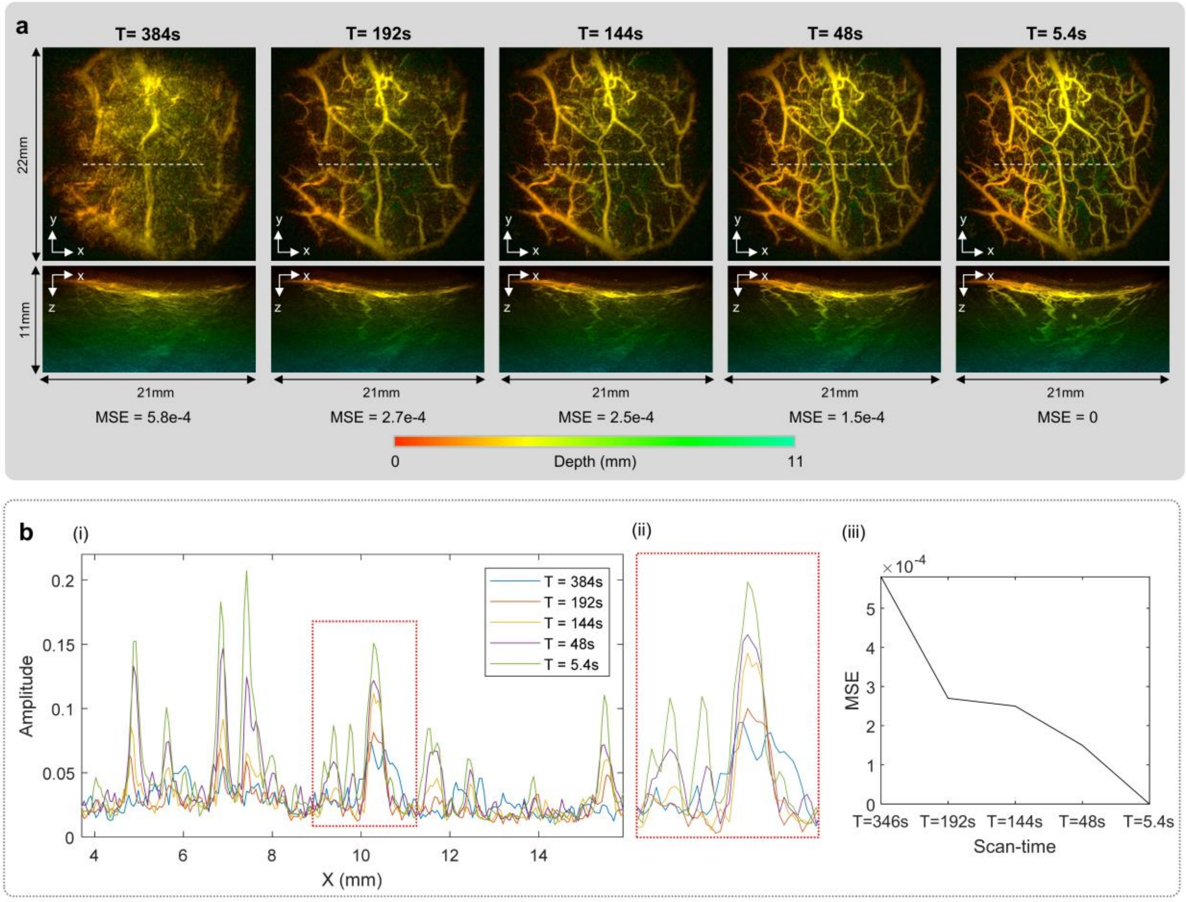

Supplementary Fig. 6| Effect of scan-time T on *in vivo* images of the palm of a healthy volunteer. (a) x-y and x-z depth-to-colour encoded MIPs. (b) (i) Horizontal profiles acquired (following image registration) at the position indicated by the horizontal dotted white line in the x-y MIPs. (ii) Expanded view of region indicated by red dashed rectangle in (i). (iii) Mean square error (MSE) versus T. Imaging parameters: λ=875nm, dx=dy=108µm, dt=16.67ns.

To acquire the above images, 106 frames were acquired using the 64 beam scanner and a 100Hz PRF over a total period of 730 s. Data was then extracted from this master data set to synthesise the scan-times of notional* early generation single[7] and 8-beam[8] FP scanners. The scan-times T corresponding to each instrument configuration are specified in the table below.

T (s)	Scanner configuration	PRF (Hz)
384	Single beam	100
192	8-beam	25
144	8-beam	33
48	8-beam	100
5.4	64-beam	100

The images and profiles above show that a notional single beam scanner providing a scan-time T=384s results in limited image quality. Even the largest vessels are poorly resolved due to motion-induced blurring and distortion. Image quality is improved with the shorter scan times achievable with 8-beam read-out but remains significantly lower than the 64 beam scanner in all cases. Even at 100Hz (T=48s) where the visual appearance of the image is improved, inspection of the profiles show that the image contrast and visibility of the smallest vessels are compromised compared to the 64 beam scanner (T=5.4s). It should be noted that these results were obtained under a best case

* The term "notional" is used here because the scan-times T are based on the assumption that each scanner configuration in the above table can provide PRF limited A-line rates. In practice, hardware latencies and other factors often meant that early generation FP scanners were unable to provide PRF limited acquisition.

scenario with a young healthy volunteer able to maintain a high level of positional stability. In clinical contexts where it may be required to scan unwell or elderly patients or parts of the body subject to greater motion, the long scan times associated with early generation FP scanners can be expected to result in substantially greater image degradation.

Supplementary Note 1

Fabry Perot ultrasound sensor modelling

Modelling the performance of the Fabry Perot (FP) ultrasound sensor requires consideration of the acoustically-induced change in its optical thickness and the conversion of that thickness change to a modulation in the reflected optical power.

The transduction mechanism can be described with reference to the FP interferometer transfer function (ITF), which represents the optical power P reflected from the sensor as a function of wavelength λ. An incident acoustic wave modulates the optical thickness of the sensor, producing a time-varying wavelength shift dλ in the ITF. When the interrogation laser wavelength λ_b is aligned at a point on the ITF where the slope is non zero, dλ is converted to a modulation in the power dP reflected from the sensor.

The optical sensitivity I_s is then given by the derivative of the ITF at λ_b. It represents the reflected optical power change dP per unit acoustically-induced ITF wavelength shift dλ:

$$I_s = \left[\frac{\mathrm{dP}}{\mathrm{d}\lambda}\right]_{\lambda_b}$$

The acoustic sensitivity A_s is the ITF wavelength shift produced per unit acoustic pressure dp:

$$A_s = \left[\frac{\mathrm{d}\lambda}{\mathrm{dp}}\right]$$

The overall sensitivity is the reflected optical power modulation per unit acoustic pressure. It is thus the product of I_s and A_s

$$S = \left[\frac{\mathrm{dP}}{\mathrm{dp}}\right] = I_s\,.\,A_s$$

Modelling the optical sensitivity requires simulating the intracavity optical field for an incident Gaussian beam as a function of wavelength in order to compute the ITF. This can be achieved in several ways depending on the sensor design, fabrication method and underlying assumptions using full wave electromagnetic descriptions[9,10,11], unfolded cavity approaches[12,13], the ABCD transfer matrix method[14] and the angular Airy function[15,16]. By simulating the ITF as a function of the mirror reflectivities, interrogation beam dimensions and sensor thickness, these models can be used as predictive design tools to optimise the optical sensitivity.

Modelling the acoustic sensitivity requires describing the interaction of the incident acoustic wave with the FP sensor using an acoustic propagation model. The normally incident frequency response for negligibly thin[17] or hard dielectric FPI mirrors[18] can be simulated using analytic and numerical models respectively. A more sophisticated approach employs an elastic model to simulate the complex, frequency-dependent, directional response of the FP sensor[19,20] and provides a complete description of the acoustic response.

Supplementary Note 2

MPE calculations

According to EN-60825-1[21], for a repetitively pulsed laser emitting a pulse train with a pulse-repetition-frequency PRF, pulse durations between 10^{-9}s and 10^{-7}s and exposure time T, the maximum permissible exposure (MPE) for the skin is defined as the most restrictive of the following two MPEs[†]:

1. The single pulse MPE which is given by MPE_{SP}= 200 C_4 J/m^2
2. The average exposure MPE which is given by:
    a. T<10s, MPE_{av}= (1.1 x 10^4 C_4 $T^{0.25}$)/(PRF x T) J/m^2
    b. T>10s, MPE_{av}=2000 C_4 W/m^2

where $C_4=10^{0.002\,(\lambda-700)}$ for λ=700 nm to 1050 nm and C_4=5 for λ=1050 nm to 1400 nm.

For all experiments in this study, the average exposure MPE is the most restrictive case and thus defines the MPE.

Taking the 1kHz fibre laser operating at 1064nm as an example, C_4=5 and for the T=0.3s scan-time used to acquire the image in Fig. 5, MPE_{av}=135.7 J/m^2. For the 20mm beam diameter used, assuming a top-hat profile, the d_{63} beam diameter (as required for MPE calculations) is 16mm and hence the illuminated area is 201.1 x 10^{-6} m^2. The pulse energy was 2.7mJ resulting in a radiant exposure H=13.4 J/m^2 which is below MPE_{av}. These parameters are summarised below along with the OPO laser system parameters used and the relevant MPEs, expressed as radiant exposures.

**λ (nm)**	**PRF (Hz)**	**T(s)**	**H (J/m^2)**	**MPE_{av} (J/m^2)**
1064	1000	0.3	13.4	135.7
850	100	3.2	37	92
850	100	5.4	44	62
850	100	15	36	40
850	100	29	44	40
850	200	1.6	23	77

[†] The "pulse in a pulse train" MPE is not required for skin exposure according to EN-60825-1

Supplementary Note 3

Iterative model-based image reconstruction approach for compressed sensing data

Model-based image reconstruction methods are flexible schemes that can be used to reconstruct PAT images from compressed sensing (CS) data, in this case spatially sub-sampled measurements of the photoacoustic wavefield. They provide a way to model the physics of the ultrasonic wave propagation and the compression technique used. Furthermore, they allow *a-priori* information via constraints and regularisation that can compensate for the information that the data does not provide due to its compression. A full description of the scheme used here with further references given in reference 22.

The forward model is given by

$$f = CAp_0 + \varepsilon \tag{1}$$

where $p_0 \geq 0$ denotes the initial pressure distribution discretised over a voxel grid in space. The linear operator A models the ultrasonic wave propagation in the medium and maps p_0 to the time-dependent pressure field over the sensor area while the linear operator C models the conversion of this pressure field into noisy measurement data f (ε represents additive noise). A detailed discussion of the operator A and its adjoint A^T (used below), and their implementation using the k-Wave MATLAB Toolbox[23,29] can be found in reference 24. In this work, C is given by point sampling on the FP sensor surface, at the locations prescribed by the 64-beam sub-sampling pattern. The image reconstruction (i.e., the solution of (1)) is formulated as a minimisation problem: among all images $p_0 \geq 0$, the objective is to find the one with the best balance between matching the data and being structurally simple, an *a-priori* assumption that is represented using the total variation (TV) regularization energy:

$$\widehat{p_0} = \underset{p_0 \geq 0}{\operatorname{argmin}} \frac{1}{2} \|CAp_0 - f\|_2^2 + \lambda\, TV(p_0) \tag{2}$$

The regularisation parameter λ controls the balance between the data fidelity term and the TV energy, which is given by the L1 norm of the gradient of p_0 computed as

$$\mathrm{TV}(p_0) = \sum_{(i,j,k)} \sqrt{\left(p_{0(\boldsymbol{i+1},\boldsymbol{j},k)} - p_{0(\boldsymbol{i},\boldsymbol{j},\boldsymbol{k})}\right)^2 + \left(p_{0(\boldsymbol{i},\boldsymbol{j}+1,k)} - p_{0(\boldsymbol{i},\boldsymbol{j},k)}\right)^2 + \left(p_{0(\boldsymbol{i},\boldsymbol{j},k+1)} - p_{0(\boldsymbol{i},\boldsymbol{j},k)}\right)^2}$$

In general, the TV energy quantifies how much an image varies across pixels. Highly textured or noisy images will have a large TV energy, while smooth or piecewise constant images have a low energy. TV regularisation suppresses noise by encouraging piecewise smooth regions, yet it preserves sharp edges between them. To solve the convex but non-smooth optimization problem (2), we use iterative optimization schemes. The basic proximal gradient scheme is:

$$p_0^{k+1} = \mathrm{prox}_{TV+,\eta\lambda}\left(p_0^k - \eta A^T C^T\left(CAp_0^k - f\right)\right) \tag{3}$$

The proximal operator $\mathrm{prox}_{TV+,\eta\lambda}(p_0)$ denotes a non-negativity-constrained TV denoising, which is equation (2) with the identity in place of the CA operator. It is implemented by a primal-dual hybrid gradient algorithm as described in reference[25]. The step size η is set to $1.8/L$ where L is the largest eigenvalue of $A^T C^T CA$, a quantity that can be precomputed for a given measurement protocol. The basic scheme (3) is extended by a FISTA-type acceleration[26].

Quantitative evaluation of the effect of spatial sub-sampling on PAT image quality

Inevitably, image quality will be reduced when using sub-sampled data. To quantify the degradation, images were reconstructed for different sub-sampling factors and the CNR, mean-square-error (MSE)

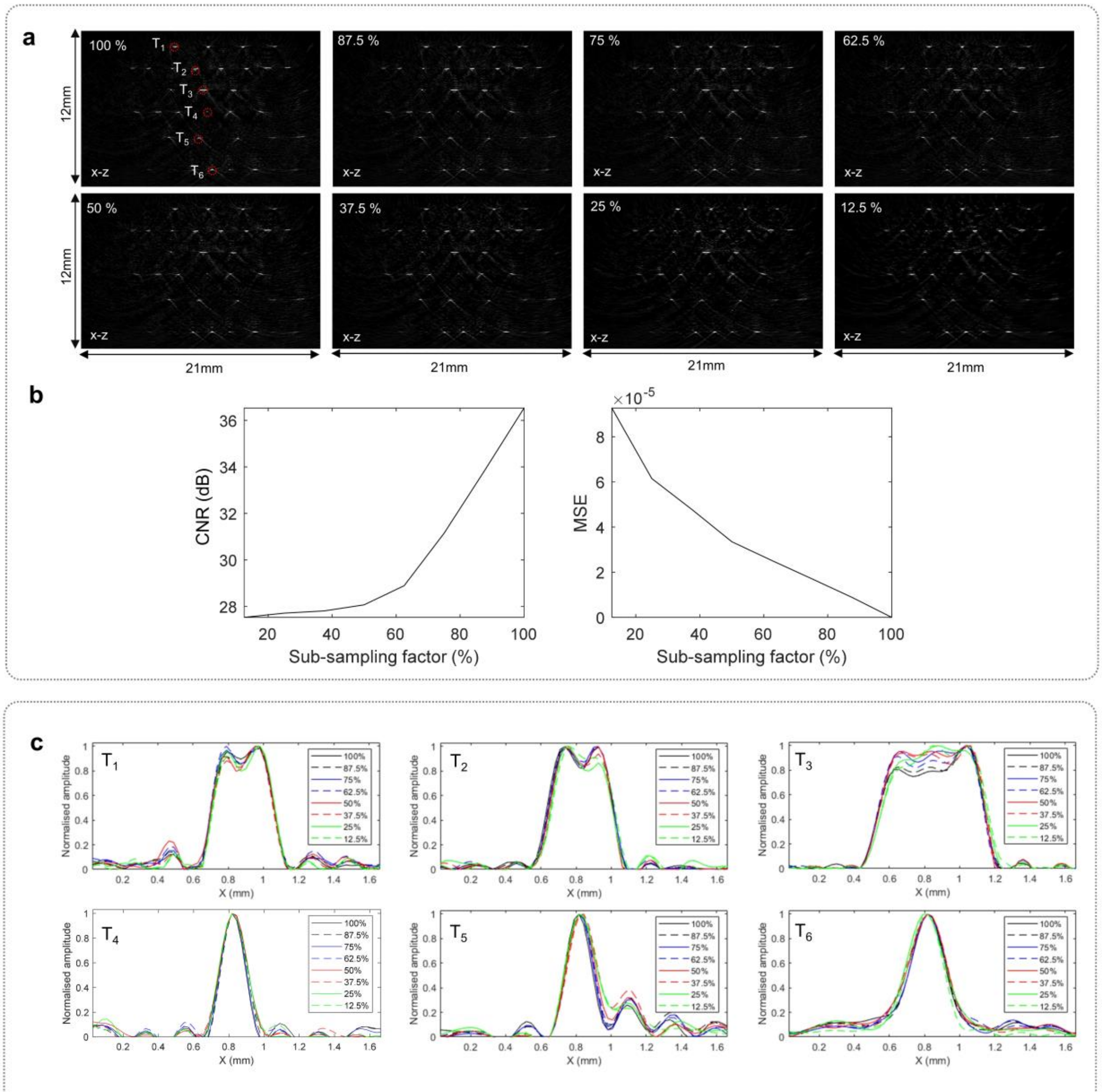

	100 %	87.50 %	75 %	62.50 %	50 %	37.50 %	25%	12.50 %
T_1	62.675	68.125	65.4	62.675	62.675	65.4	65.4	70.85
T_2	79.025	84.475	81.75	76.3	68.125	70.85	73.575	81.75
T_3	89.925	84.475	84.475	81.75	95.375	100.825	98.1	95.375
T_4	103.55	103.55	106.275	109	111.725	103.55	106.275	109
T_5	109	106.275	109	109	109	106.275	114.45	119.9
T_6	117.175	119.9	122.625	117.175	117.175	114.45	119.9	114.45

Supplementary Fig. 7| Effect of sub-sampling factor (SSF) on CNR, MSE and spatial resolution. (a) PAT images of phantom comprised of absorbing ribbons reconstructed for SSFs of 87.5%, 75%, 62.5%, 50%, 37.5%, 25% and 12.5% for λ=9e-4. (b) Dependence of image CNR and MSE on SSF. (c) Horizontal profiles through centrally located absorbing features T_1, T_2, T_3, T_4, T_5 and T_6 . The table provides the measured line-spread-function (LSF) (in microns) for each absorbing feature extracted from the rising edges of profiles. The LSF provides a measure of spatial resolution.

and spatial resolution estimated. A well defined resolution phantom composed of small absorbing ribbons distributed throughout the FOV was used for this purpose.

A fully sampled scan of the phantom was performed. The measured data was then progressively sub-sampled and images reconstructed for each sub-sampling factor (SSF), as shown in Supplementary Fig. 7a. Supplementary Fig. 7b shows that the image CNR decreases with increasing SSF; for example

it falls by 11dB for the highest SSF of 12.5% compared to the full-sampled (100%) image. The MSE, which is commonly used to assess compressed images, quantifies the extent to which the sub-sampled images differ from the fully-sampled image. It thus provides a measure of artefacts and spatial infidelities introduced by sub-sampling, as well as the noise. As Supplementary Fig. 7b shows, the MSE increases as the SSF increases.

To assess the effect on spatial resolution, horizontal profiles (Supplementary Fig. 7c) were taken through the centrally located absorbers at different depths for each SSF. The profiles overlap closely exhibiting similarly sharp edges and widths for all SSFs suggesting that any resolution degradation is modest. This is evidenced quantitatively by measurements of the line-spread-function (LSF) which were obtained by computing the derivative of the edge-spread-function (ESF) for the rising edge of each feature. The LSF serves as a measure of the spatial resolution and is provided for each centrally located absorbing feature in the table in Supplementary Fig. 7c which shows that the LSFs for all SSFs lie within 13% of the fully sampled image LSFs. This observation is consistent with expectations as TV regularisation is well known from its application in other compressed sensing imaging contexts[27] to preserve the edges of reconstructed image features.

The behaviour observed in the sub-sampled phantom images is also reflected in the structurally more diverse *in vivo* images in Fig. 5 (main manuscript) reproduced below in Supplementary Fig. 8. The quantitative analysis of these images shows that the CNR decreases and the MSE increases with increasing SSF which is also visually apparent from the two vertical profiles taken. The profiles also show that the sharpness of the edges and the lateral dimensions of the reconstructed features are similar for all SSFs. As with the phantom images, this suggests any resolution and spatial fidelity degradation with SSF is small.

In summary, for the image examples used, the main trade-off in terms of image quality is the CNR loss as the SSF is increased. In a more general sense however there is an additional trade-off that warrants consideration. This is associated with the sparsity constraint imposed by TV regularisation which limits the structural diversity that can be tolerated before image quality is compromised by excessive noise, as discussed in references 24 and 28. However, for PAT images of predominantly vascular anatomy, the structural diversity is relatively low and so this trade-off is manageable.

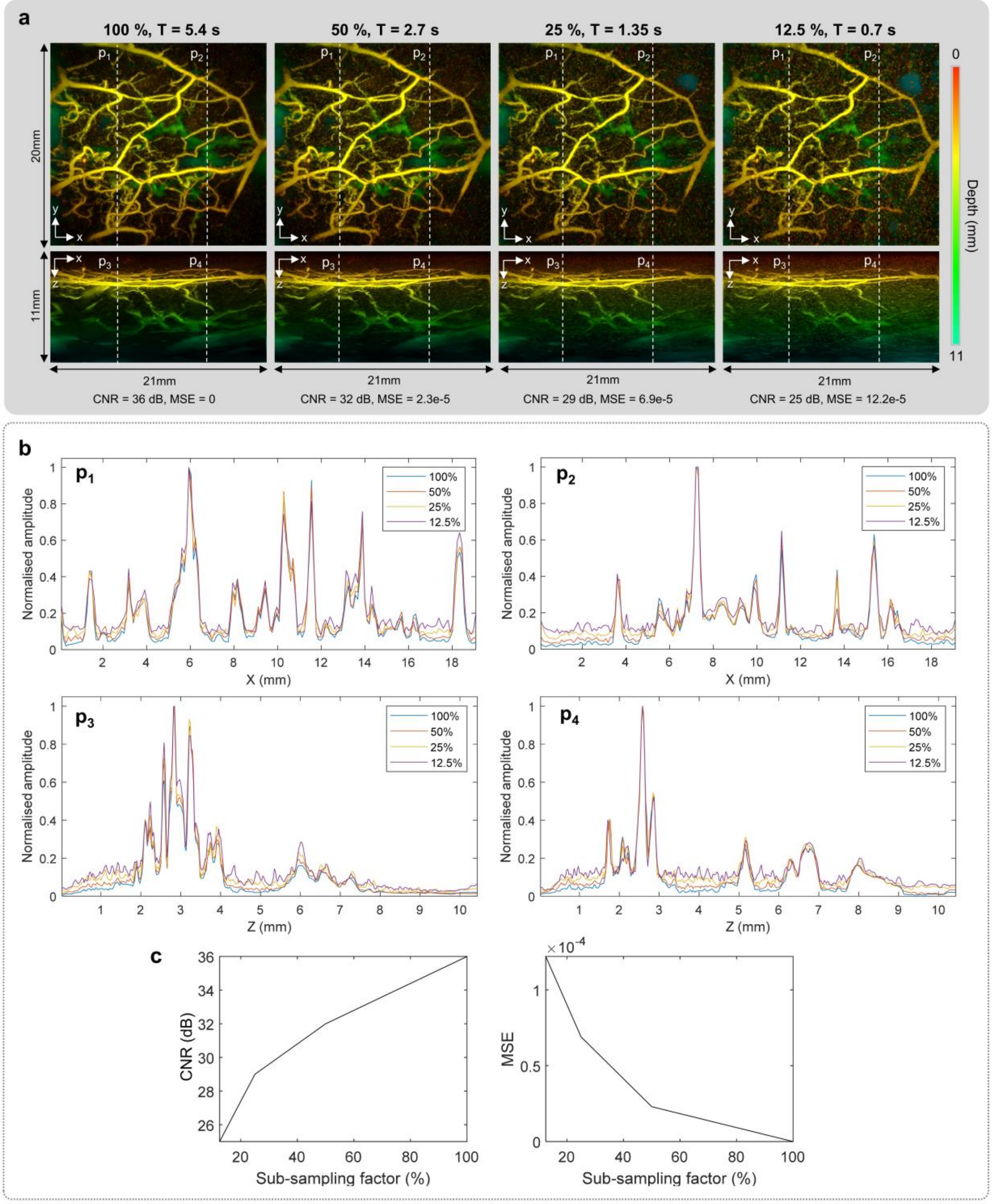

Supplementary Fig. 8| *In vivo* sub-sampled PAT images reproduced from Fig. 5b in main paper. Horizontal and vertical Profiles (normalised to peak image intensity) p_1, p_2, p_3, p_4 illustrating the reduction in CNR and increase in MSE with SSF whilst preserving the edges and widths of the reconstructed blood vessels.

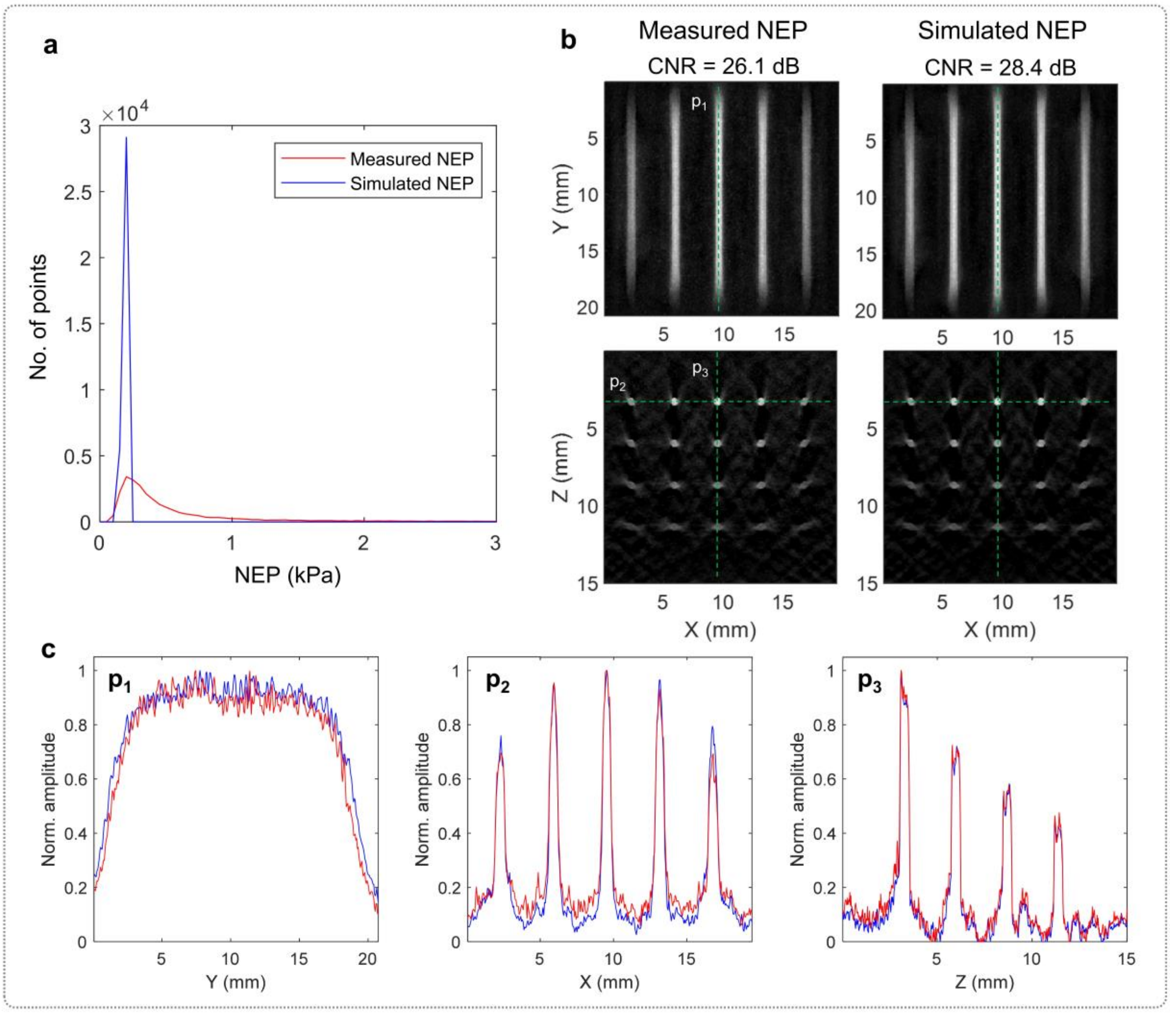

Supplementary Fig. 9| Effect of NEP distribution on image quality. (a) NEP histograms. The red line is the experimentally measured NEP distribution for the 64 beam scanner shown in Fig. 1e (main manuscript); modal NEP=0.2 kPa and FWHM=0.25 kPa. The blue line is a simulated NEP distribution that represents an near-idealised hypothetical FP scanner with a narrow NEP distribution; modal NEP=0.2 kPa, FWHM=0.022 kPa. (b) Simulated photoacoustic images (MIPs) for the two cases in (a). (c) Profiles p_1, p_2 and p_3 (normalised to peak amplitude) corresponding to the dotted green lines in (b).

The simulated images were produced using k-wave[29] using the same parameters as the experimentally acquired images in Fig. 2 (main manuscript): dt = 16.67ns, dx=dy=108um and a 20.7 x 19.4 mm^2 scan area. Photoacoustic waves were generated from 20 equally spaced cylindrical absorbers and recorded at 34,560 equally spaced detection points distributed over a planar surface representing the FP sensor. Each detection point has the same sensitivity in the k-wave simulation. Hence to represent the two NEP distributions shown in Supplementary Fig. 9a, each NEP distribution was firstly normalised and then mapped on to the detection points distributed over the sensor surface in order to appropriately scale the recorded photoacoustic time-series data. Noise (10% of the peak signal amplitude) was then added to each photoacoustic time-series resulting in SNRs in the range 7:1 to 40:1 which is representative of the measured *in vivo* photoacoustic signals in this study. The signals were then filtered with a 50kHz – 20 MHz bandpass filter. Following x2 upsampling, the images were reconstructed using the k-space algorithm referred to in the Methods (main manuscript)[30]. The profiles p_1 and p_2 are taken from a 1mm thick slice (z=2.75mm to 3.75mm) and p_3 a 1.1mm thick slice (Y= 9.8mm to 10.9mm) for the locations indicated by the dotted green lines in Supplementary Fig. 9b.

Visual inspection of the reconstructed images in Supplementary Fig. 9b suggests the image quality is similar for the two NEP distributions. The main impact of the broader measured NEP distribution is

to reduce the image CNR by 2.3 dB relative to the simulated NEP distribution. The profiles in Supplementary Fig 9b suggests the spatial resolution and fidelity are similar for the two cases.

Videos

Supplementary Video 1: 2D dynamic PAT imaging of the wrist region at 33 fps (offline reconstruction). The images were reconstructed using three different methods. Unfocussed: the photoacoustic time-series from a single row of detection points was used. Sum: photoacoustic time-series from four rows were summed, Focussed: synthetic receiving focussing in the elevational plane using photoacoustic time-series from four rows was performed.

Supplementary Video 2: 2D dynamic PAT imaging of radial artery at 33 fps (offline reconstruction)

Supplementary Video 3: 2D dynamic PAT imaging of wrist region at 25 fps (online reconstruction). Pulsating radial artery can be seen from t=34s onwards

Supplementary Video 4: 3D dynamic imaging as probe is moved across finger-tip at 16.7 fps (refresh frame rate) using 10% sliding window (offline reconstruction).

Supplementary Video 5: 3D dynamic imaging as probe is moved across the palm at 3 fps (refresh frame rate) using 50% sliding window (online reconstruction).

Supplementary Video 6: 3D dynamic imaging of perfusion following arterial occlusion at 16.7 fps (refresh frame rate) using 10% sliding window (offline reconstruction).

Supplementary Video 7: 2D dynamic multiwavelength imaging.

Supplementary Video 8: Step sequence of *in vivo* 3D Images acquired at 4 different wavelengths shown in Supplementary Fig. 5.

---

[1] Jacques S., “Optical absorption of melanin,” https://omlc.org/spectra/melanin/
[2] Jacques S., "Optical Absorption of Hemoglobin"*https://omlc.org/spectra/hemoglobin/*
[3] Li M, Tang Y, Yao J. Photoacoustic tomography of blood oxygenation: A mini review. Photoacoustics. 2018 May 31;10:65-73
[4] Cox, B., Laufer, J. G., Arridge, S. R., & Beard, P. C. (2012). Quantitative spectroscopic photoacoustic imaging: a review. Journal of Biomedical Optics, 17(6), 061202.
[5] Hochuli, R., An, L., Beard, P. C., & Cox, B. T. (2019). Estimating blood oxygenation from photoacoustic images: can a simple linear spectroscopic inversion ever work? Journal of Biomedical Optics, 24(12), 1.
[6] Laufer, J., Delpy, D., Elwell, C., & Beard, P. (2007). Quantitative spatially resolved measurement of tissue chromophore concentrations using photoacoustic spectroscopy : application to the measurement of blood oxygenation and haemoglobin concentration. Physics in Medicine and Biology, 52(1), 141–168.
[7] Jathoul, A. P., Laufer, J., Ogunlade, O., Treeby, B., Cox, B., Zhang, E., … Beard, P. (2015). Deep in vivo photoacoustic imaging of mammalian tissues using a tyrosinase-based genetic reporter. Nature Photonics, 9(April), 239–246.
[8] Plumb, A. A., Huynh, N. T., Guggenheim, J., Zhang, E., & Beard, P. (2018). Rapid volumetric photoacoustic tomographic imaging with a Fabry-Perot ultrasound sensor depicts peripheral arteries and microvascular vasomotor responses to thermal stimuli. European Radiology, 28(3), 1037–1045.
[9] Marques, D. M., Guggenheim, J. A., Ansari, R., Zhang, E. Z., Beard, P. C., & Munro, P. R. T. (2020). Modelling Fabry-Pérot etalons illuminated by focussed beams. Optics Express, 28(5), 7691–7706.
[10] Marques, D. M., Guggenheim, J. A., & Munro, P. R. T. (2021). Analysing the impact of non-parallelism in Fabry-Perot etalons through optical modelling. Optics Express, 29(14), 21603.
[11] Marques, D., Guggenheim, J., & Munro, P. (2022). Numerical model of light propagation through Fabry-Perot etalons composed of interfaces with non-planar surface topography. Optics Express, 30(26), 46294–46306.

[12] Czuchnowski, J., & Prevedel, R. (2021). Improving the Sensitivity of Planar Fabry–Pérot Cavities via Adaptive Optics and Mode Filtering. Advanced Optical Materials, 9(4), 1–12.
[13] Czuchnowski, J., & Robert, P. (2022). Transfer function asymmetry in Fabry – Perot-based optical pressure sensors. Optics Letters, 47(23), 6089.
[14] Martin-Sanchez, D., Li, J., Marques, D., Zhang, E., Munro, P., Beard, P., & Guggenheim, J. (2022). ABCD transfer matrix model of Gaussian beam propagation in Fabry-Perot etalons. Optics Express, 30(26), 46404–46417. 3
[15] Marques, D. M., Guggenheim, J. A., & Munro, P. R. T. (2021). Angular Airy function: a model of Fabry-Perot etalons illuminated by arbitrary beams. Optics Express, 29(15), 24144.
[16] Lee, J. Y., Hahn, J. W., & Lee, H.-W. (2002). Spatiospectral transmission of a plane-mirror Fabry-Perot interferometer with nonuniform finite-size diffraction beam illuminations. Journal of the Optical Society of America. A, Optics, image science, and vision,19, 973-984.
[17] Beard, P. C., Perennes, F., & Mills, T. N. (1999). Transduction mechanisms of the Fabry-Perot polymer film sensing concept for wideband ultrasound detection. IEEE Transactions on Ultrasonics, Ferroelectrics and Frequency Control, 46(6), 1575–1582.
[18] Buchmann, J., Guggenheim, J., Zhang, E., Scharfenorth, C., Spannekrebs, B., Villringer, C., & Laufer, J. (2017). Characterization and modeling of Fabry–Perot ultrasound sensors with hard dielectric mirrors for photoacoustic imaging. Applied Optics, 56(17), 5039.
[19] Cox, B. T., & Beard, P. C. (2007). The frequency-dependent directivity of a planar Fabry-Perot polymer film ultrasound sensor. IEEE Transactions on Ultrasonics, Ferroelectrics, and Frequency Control, 54(2), 394–404.
[20] Ramasawmy, D. R., Martin, E., Guggenheim, J. A., Zhang, E. Z., Beard, P. C., Treeby, B. E., & Cox, B. T. (2019). Analysis of the Directivity of Glass-Etalon Fabry–Pérot Ultrasound Sensors. IEEE Transactions on Ultrasonics, Ferroelectrics, and Frequency Control, 66(9), 1504–1513.
[21] EN-60825-1 (BS EN 60825-1:2014+A11:2021, "Safety of laser products. Equipment classification and requirements"
[22] Arridge, S. R., Beard, P., Betcke, M. M., Cox, B., Huynh, N., Lucka, F., … Zhang, E. (2016). Accelerated High-Resolution Photoacoustic Tomography via Compressed Sensing. Phys Med Biol, 61, 8908–8940.
[23] Treeby, B. E., Jaros, J., & Cox, B. T. (2016). Advanced photoacoustic image reconstruction using the k-Wave toolbox. In A. A. Oraevsky & L. V. Wang (Eds.), Proceedings of SPIE (Vol.) 9708, p. 97082P.
[24]Arridge, S. R., Betcke, M. M., Cox, B. T., Lucka, F., & Treeby, B. E. (2016). On the adjoint operator in photoacoustic tomography. Inverse Problems, 32(11), 115012.
[25] Chambolle, A., & Pock, T. (2011). A First-Order Primal-Dual Algorithm for Convex Problems with Applications to Imaging. Journal of Mathematical Imaging and Vision, 40(1), 120–145
[26] Beck, Teboulle, 2009, Fast gradient-based algorithms for constrained total variation image denoising and deblurring problem]
[27] Benning, M., & Burger, M. (2018). Modern regularization methods for inverse problems. Acta Numerica, 27, 1–111.
[28] Zhu, J., Huynh, N., Ogunlade, O., Ansari, R., Lucka, F., Cox, B., & Beard, P. (2023). Mitigating the Limited View Problem in Photoacoustic Tomography for a Planar Detection Geometry by Regularized Iterative Reconstruction. IEEE Transactions on Medical Imaging, 42(9), 2603–2615.
[29] Treeby, B. E., & Cox, B. T. (2010). k-Wave: MATLAB toolbox for the simulation and reconstruction of photoacoustic wave fields. Journal of Biomedical Optics, 15(2), 021314
[30] Köstli, K. P., Frenz, M., Bebie, H., & Weber, H. P. (2001). Temporal backward projection of optoacoustic pressure transients using Fourier transform methods. Physics in Medicine and Biology, 46(7), 1863–1872.
