# Supplementary material for: A fast all-optical 3D photoacoustic scanner for clinical vascular imaging
Source: Nat Biomed Eng. 2024 Sep 30;9(5):638–55. doi: 10.1038/s41551-024-01247-x (PMC12092260; doi:10.1038/s41551-024-01247-x)
Supplement: Supplementary file 2 — Reporting Summary [file 41551_2024_1247_MOESM2_ESM.pdf]

nature portfolio

Corresponding author(s): Paul Beard

Last updated by author(s): Jul 2, 2024

Reporting Summary

Nature Portfolio wishes to improve the reproducibility of the work that we publish. This form provides structure for consistency and transparency in reporting. For further information on Nature Portfolio policies, see our Editorial Policies and the Editorial Policy Checklist.

Statistics

For all statistical analyses, confirm that the following items are present in the figure legend, table legend, main text, or Methods section.

n/a	Confirmed	
☐	☒	The exact sample size (*n*) for each experimental group/condition, given as a discrete number and unit of measurement
☐	☒	A statement on whether measurements were taken from distinct samples or whether the same sample was measured repeatedly
☐	☒	The statistical test(s) used AND whether they are one- or two-sided<br>*Only common tests should be described solely by name; describe more complex techniques in the Methods section.*
☒	☐	A description of all covariates tested
☒	☐	A description of any assumptions or corrections, such as tests of normality and adjustment for multiple comparisons
☐	☒	A full description of the statistical parameters including central tendency (e.g. means) or other basic estimates (e.g. regression coefficient) AND variation (e.g. standard deviation) or associated estimates of uncertainty (e.g. confidence intervals)
☐	☒	For null hypothesis testing, the test statistic (e.g. *F*, *t*, *r*) with confidence intervals, effect sizes, degrees of freedom and *P* value noted<br>*Give P values as exact values whenever suitable.*
☒	☐	For Bayesian analysis, information on the choice of priors and Markov chain Monte Carlo settings
☒	☐	For hierarchical and complex designs, identification of the appropriate level for tests and full reporting of outcomes
☒	☐	Estimates of effect sizes (e.g. Cohen's *d*, Pearson's *r*), indicating how they were calculated

Our web collection on statistics for biologists contains articles on many of the points above.

Software and code

Policy information about availability of computer code

Data collection	The photoacoustic image datasets were collected using custom Labview 2017 (64 bit) acquisition software
Data analysis	All image datasets were processed and analysed using custom Matlab (R2017b and R2020b.) or C++ scripts. They are are available from the authors for research purposes on reasonable request.

For manuscripts utilizing custom algorithms or software that are central to the research but not yet described in published literature, software must be made available to editors and reviewers. We strongly encourage code deposition in a community repository (e.g. GitHub). See the Nature Portfolio guidelines for submitting code & software for further information.

Data

Policy information about availability of data

All manuscripts must include a data availability statement. This statement should provide the following information, where applicable:

- Accession codes, unique identifiers, or web links for publicly available datasets
- A description of any restrictions on data availability
- For clinical datasets or third party data, please ensure that the statement adheres to our policy

The raw image datasets and reconstructed-image data are available from the authors for research purposes on reasonable request.

Research involving human participants, their data, or biological material

Policy information about studies with human participants or human data. See also policy information about sex, gender (identity/presentation), and sexual orientation and race, ethnicity and racism.

Reporting on sex and gender	20 participants in total were recruited: 8 heathy volunteers (5 male, 3 female) and 12 patients (4 male, 8 female). Sex and gender were not considered in the study design.
Reporting on race, ethnicity, or other socially relevant groupings	The participants were not categorized according to race, ethnicity or any other groupings.
Population characteristics	See above.
Recruitment	The volunteers were recruited from the Department of Medical Physics and Biomedical Engineering, UCL. Patients were recruited from UCL Hospitals NHS trust. Written informed consent was obtained from all participants.
Ethics oversight	Ethical permission was granted by University College London Research Ethics Committee (Project ID: 1133/001) for the volunteer studies. Ethical permission was granted by NHS Research Ethics Committee approval (IRAS Project ID number: 206196) for the patient studies.

Note that full information on the approval of the study protocol must also be provided in the manuscript.

Field-specific reporting

Please select the one below that is the best fit for your research. If you are not sure, read the appropriate sections before making your selection.

☒ Life sciences ☐ Behavioural & social sciences ☐ Ecological, evolutionary & environmental sciences

For a reference copy of the document with all sections, see nature.com/documents/nr-reporting-summary-flat.pdf

Life sciences study design

All studies must disclose on these points even when the disclosure is negative.

Sample size	15 healthy volunteers and 16 patients were recruited. No sample-size calculation was performed, as the study was largely focused on technology evaluation and was designed as an initial technical proof-of-concept.
Data exclusions	Imaging data was acquired on multiple occasions on most participants, but only one representative image is typically shown for each anatomical location or clinical condition, to illustrate technical proof-of-concept.
Replication	Data acquisition was performed on most participants multiple times to ensure correct device functioning. All attempts at replication were successful.
Randomization	The study is a technical proof-of-concept, so randomization was not relevant.
Blinding	The investigators were not blinded to the study. Blinding was not relevant to the study owing to its technical proof-of-concept nature.

Reporting for specific materials, systems and methods

We require information from authors about some types of materials, experimental systems and methods used in many studies. Here, indicate whether each material, system or method listed is relevant to your study. If you are not sure if a list item applies to your research, read the appropriate section before selecting a response.

Materials & experimental systems

n/a	Involved in the study	
☒	☐	Antibodies
☒	☐	Eukaryotic cell lines
☒	☐	Palaeontology and archaeology
☒	☐	Animals and other organisms
☒	☐	Clinical data
☒	☐	Dual use research of concern
☒	☐	Plants

Methods

n/a	Involved in the study	
☒	☐	ChIP-seq
☒	☐	Flow cytometry
☒	☐	MRI-based neuroimaging
